# Movement Disorders and Liver Disease

**DOI:** 10.1002/mdc3.13238

**Published:** 2021-05-31

**Authors:** Eoin Mulroy, Francesca Baschieri, Francesca Magrinelli, Anna Latorre, Pietro Cortelli, Kailash P. Bhatia

**Affiliations:** ^1^ Department of Clinical and Movement Neurosciences UCL Queen Square Institute of Neurology London United Kingdom; ^2^ IRCCS Istituto delle Scienze Neurologiche di Bologna Bologna Italy; ^3^ Dipartimento di Scienze Biomediche e Neuromotorie Università di Bologna Bologna Italy; ^4^ Department of Neurosciences Biomedicine and Movement Sciences, University of Verona Verona Italy

**Keywords:** liver diseases, movement disorders

## Abstract

The association of movement disorders with structural or functional hepatic disease occurs in three principal scenarios: (1) combined involvement of both organ systems from a single disease entity, (2) nervous system dysfunction resulting from exposure to toxic compounds in the setting of defective hepatic clearance, or (3) hepatic and/or neurological injury secondary to exposure to exogenous drugs or toxins. An important early step in the workup of any patient with combined movement disorders and liver disease is the exclusion of Wilson's disease. Diagnostic delay remains common for this treatable disorder, and this has major implications for patient outcomes. Thereafter, a structured approach integrating variables such as age of onset, tempo of progression, nature and severity of liver involvement, movement disorder phenomenology, exposure to drugs/toxins and laboratory/neuroimaging findings is key to ensuring timely diagnosis and disease‐specific therapy. Herein, we provide an overview of disorders which may manifest with a combination of movement disorders and liver disease, structured under the three headings as detailed above. In each section, the most common disorders are discussed, along with important clinical pearls, suggested diagnostic workup, differential diagnoses and where appropriate, treatment considerations.

The association of movement disorders (MD) with hepatic disease is a noteworthy one. In some instances, it is merely coincidental. In others, it may reflect:

1. Combined involvement of both organ systems from a single disease entity,
2. Neurological dysfunction secondary to defective hepatic clearance of toxic compounds, or
3. Hepatic and/or neurological dysfunction secondary to exposure to exogenous drugs or toxins.

A structured approach considering variables such as patient age, duration and severity of liver disease, MD phenomenology and ancillary tests can facilitate diagnosis, and oftentimes, institution of specific treatment. In this review, we provide an educational and practical overview of MD associated with hepatic disease. We begin with an overview of liver disease and its diagnostic workup, and proceed to discuss the three categories of disorders detailed above. Common conditions are discussed in the text, while less frequent associations are detailed in Tables S1 and S2. An approach to diagnosis is suggested in Figure 1. Our methods are described in supplementary material S3.

**FIG. 1:**
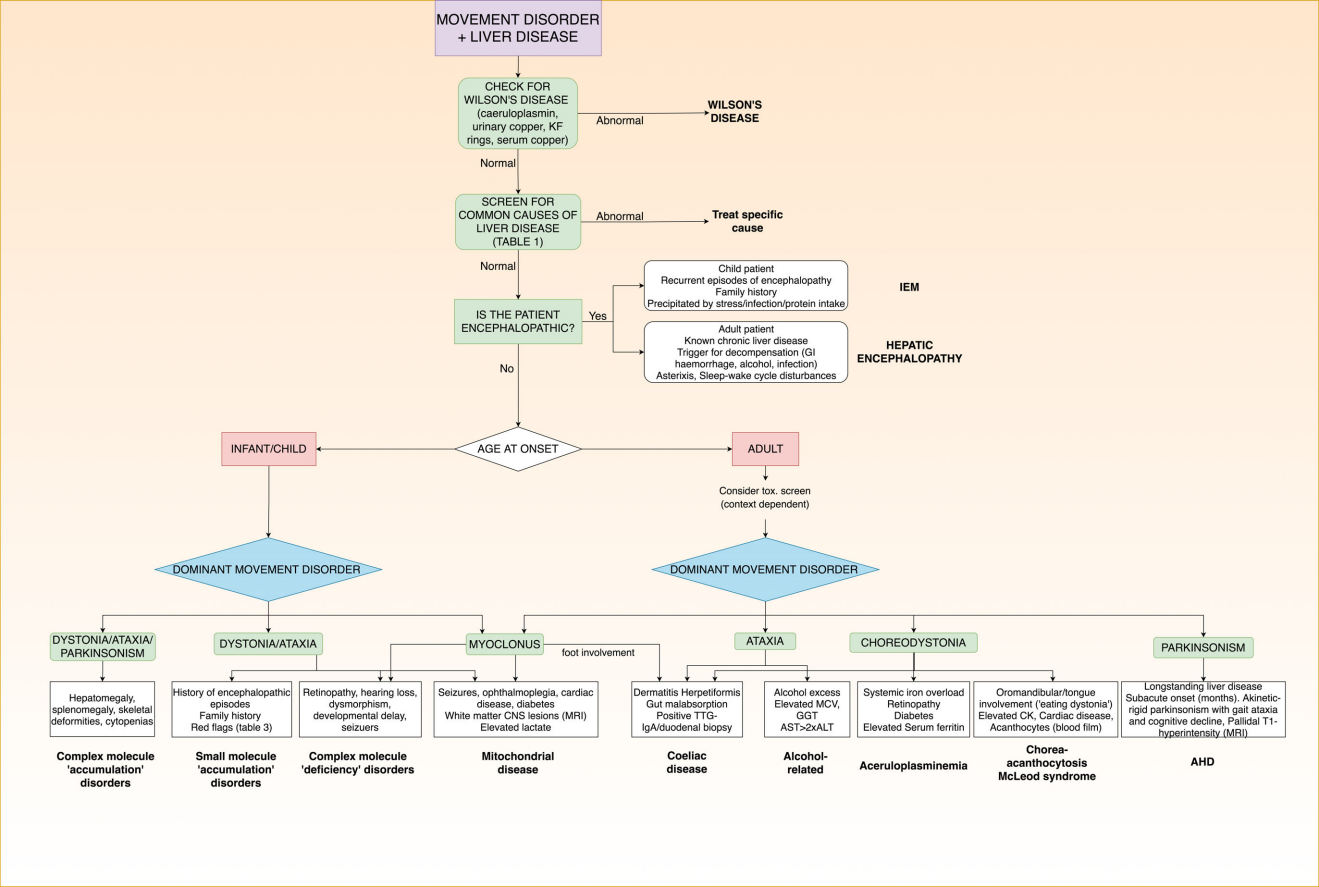
Simple diagnostic algorithm for patients presenting with movement disorders + liver disease. (AHD: acquired hepatocerebral degeneration; AST: aspartate aminotransferase; ALT: alanine transaminase; CK: creatine kinsase; CNS: central nervous system; IEM: inborn error of metabolism; KF: Kayser‐Fleischer; MCV: mean cell volume; MRI: magnetic resonance imaging; TTG: tissue transglutaminase).

## A Brief Overview of Liver Disease

Liver disease is a major cause of premature mortality worldwide. Its prevalence is increasing, largely driven by alcoholic and non‐alcoholic fatty liver disease, and viral hepatitis. Liver dysfunction is frequently asymptomatic or produces non‐specific complaints such as asthenia or malaise. Occasionally, more specific abnormalities‐jaundice, pruritis, bleeding diathesis, immunosuppression, oedema‐may emerge. Clinical examination can identify other suggestive features, including palmar erythema, spider naevi, ascites and hepatomegaly.

Liver dysfunction is most commonly identified through testing a panel of serum markers, commonly referred to as “liver function tests”.^1^ These comprise markers of hepatocellular dysfunction‐alanine transaminase (ALT) and/or aspartate aminotransferase (AST), bilirubin—a marker of parenchymal liver dysfunction or biliary obstruction, and markers of biliary disease such as alkaline phosphatase(ALP) and gamma—glutamyltransferase (GGT). Further, other indices eg, albumin and INR, are useful in assessing hepatic synthetic function. However, most are not specific for liver disease. For instance, AST may increase following muscle injury, elevations in GGT are common in chronic alcoholics, obese individuals or those taking enzyme‐inducing drugs, and ALP can be deranged in metabolic, structural or malignant bone disease, and during childhood and pregnancy. These tests must therefore always be interpreted in the clinical context.

Liver injury progresses through a number of stages, beginning with steatosis (accumulation of fat). This relatively “benign” process is often reversible if its primary driver (e.g. alcohol, metabolic syndrome) is addressed.^2^ Up to half of people with steatosis develop hepatic inflammation (steatohepatitis)—many of these will progress to fibrosis/cirrhosis.^2^ Most cirrhotic patients exist in a “compensated” state, with decompensations, marked by the development of ascites, jaundice, encephalopathy or variceal hemorrhage, occurring in the setting of gastrointestinal bleeding, infection or continued insults eg, alcohol ingestion.

In addition to defining etiology (Table 1), chronic liver disease patients should undergo assessments for liver fibrosis, which is a strong predictor of future morbidity and mortality.^3^ Blood biomarkers (often combined with clinical/demographic data) or imaging techniques (transient elastography) are frequently employed, though the gold standard remains liver biopsy.

**TABLE 1:** Common first‐line screening examinations in adults with abnormal liver function tests (LFTs)

Screening test	Etiology
Serum copper, caeruloplasmin AND Urinary copper. Slit‐lamp examination	Wilson's disease
Hepatitis B surface antigen, hepatitis C antibody	Viral hepatitis
Ferritin, transferrin saturation, serum iron, total iron binding capacity	Hereditary Haemochromatosis
Tissue transglutaminase IgA	Coeliac disease
Alcohol history (+biochemical clues: ↑MCV, ↑GGT, AST > 2xALT)	Alcoholic liver disease
↑BMI, ↑haemoglobin a1c. Lipid profile. Liver ultrasound or MRI	Non‐alcoholic steatosis/steatohepatitis
Anti‐mitochondrial antibody, anti‐smooth muscle antibody, Anti‐LKM antibody, antinuclear antibody, serum immunoglobulins	Autoimmune liver disease
Alpha‐1 antitrypsin	Alpha‐1 antitrypsin deficiency

ALT, alanine transaminase; AST, aspartate aminotransferase; BMI, body mass index; GGT, gamma‐glutamyltransferase; LKM, Liver‐Kidney microsomal; MCV, mean cell volume; MRI, magnetic resonance imaging.

The complexity of liver function testing, detecting hepatic fibrosis and managing complications mandates the early involvement of a hepatologist in cases of combined MD and hepatic disease.

## Combined Involvement of the Nervous System and Liver from a Single Disease Entity

This category largely comprises genetic disorders which manifest in childhood or early adolescence. MD phenomenology is varied, but frequently dystonic or ataxic in nature. Liver involvement ranges from mild/asymptomatic, through to fulminant liver failure. Hepatomegaly is a particular red flag for storage disorders and other inborn errors of metabolism.

### Wilson's Disease

Wilson's disease (WD) is the archetypal disorder combining MD and liver disease. Characterized by the triad of liver disease, neurological abnormalities and psychiatric symptoms, this recessively inherited condition results from biallelic mutations in *ATP7B*, coding for a copper‐transporting ATPase. The resulting defective biliary copper excretion results in systemic copper overload and end‐organ toxicity.^4^

Most individuals manifest in the 1st–3rd decades of life. Age and sex are important determinants of disease phenotype, while *ATP7B* genotype is an important determinant of penetrance.^5^, ^6^ Hepatic manifestations often predominate in childhood, while neurological presentations occur more frequently in adolescence/early adulthood.^4^, ^5^

Hepatic manifestations of WD are protean, existing along a spectrum ranging from completely undetectable through varying degrees of chronic active hepatitis, progressing if untreated to cirrhosis and decompensated liver disease.^7^ Acute hepatic failure is the presenting feature in roughly 5% of cases and carries 100% mortality without urgent liver transplant—it is particularly common in young women.^8^, ^9^, ^10^, ^11^ In such cases of acute hepatic failure, Coombs‐negative intravascular haemolysis (which itself can be a presenting feature of WD) is an important clinical clue. Acute‐on‐chronic liver failure can also occur following sudden discontinuation of therapy.^8^, ^9^

Neurological manifestations are equally varied, but generally dominated by MD. Kinnier Wilson asserted in his landmark paper that tremor was one of the “outstanding features” of WD, and this has stood the test of time.^12^ It can be resting, postural (including the classic “wing‐beating”), intention or action tremor and affects up to 90% of patients with neurological WD at some time point.^13^ Though generally first appearing in the upper limbs, if untreated it may progress to involve the trunk, legs and head.^13^ Dystonia is also common, often affecting the facial musculature, producing a “fatuous smile”; again, without treatment, axial and limb involvement ensues.^12^, ^13^, ^14^ Parkinsonism is observed in roughly 30%, while chorea and ataxia may also be seen. Dysarthria, the most common feature of neurological WD, is present in almost all cases.^13^, ^14^ Psychiatric disturbances(personality and behavioral changes, psychosis) and cognitive impairment often associate with MD.^15^ MRI abnormalities including basal ganglia, thalamus and brainstem T2‐weighted hyperintensities and in more advanced cases, the typical “face of the giant panda” or “face of the panda cub” signs may be apparent.^14^, ^16^

The approach to diagnosis of WD depends on the clinical presentation, as the sensitivity and specificity of diagnostic tests vary in different scenarios. Numerous guidelines have been published to aid clinicians in this regard.^17^, ^18^ In symptomatic individuals, diagnosing WD largely relies on clinical and/or biochemical identification of systemic copper overload, through demonstration of elevated urinary copper excretion, reduced serum caeruloplasmin, ocular Kayser‐Fleischer rings or in some cases, copper overload on liver biopsy.

Clinicians should be mindful of pitfalls in the interpretation of these screening tests for WD. Kayser‐Fleischer rings are present in only 50% of patients with hepatic WD, but 85%–95% of those with neuropsychiatric involvement.^8^ Caeruloplasmin levels may be reduced in protein‐losing nephropathies and enteropathies and in advanced liver disease of any cause. Cholestatic liver disease can cause elevated urinary copper excretion, and occasionally, Kayser‐Fleischer rings.^18^ Moreover, in fulminant hepatic failure due to WD, classic approaches to testing, in particular serum caeruloplasmin are both insensitive and non‐specific. Additionally, reduced urine output alongside processing delays limits the utility of urine copper excretion in this setting.^10^ This diagnosis therefore requires recognition of other biochemical features, classically modest transaminase elevations, low ALP and ratio of ALP:bilirubin of <2.^10^, ^18^ In children with hepatic WD, caeruloplasmin and urinary copper elimination may be misleadingly normal, and liver biopsy may be necessary to establish the diagnosis.^19^

Though the *ATP7B* gene was identified in 1993, genetic heterogeneity, incomplete penetrance and the presence of modifier genes limit the utility of genetic screening as a diagnostic test in symptomatic WD. Genetic screening alone will miss the diagnosis in 15%–20% of cases.^5^, ^20^ Moreover, homozygosity or compound heterozygosity for pathogenic *ATP7B* variants does not imply that disordered copper metabolism, and hence WD, will ensue.^20^ Hence, confirmatory biochemical testing is always required in the presence of positive genetic testing and treatment decisions should be based on clinical and biochemical rather than genetic parameters. Genetic testing is useful as a confirmatory test, and for screening of family members.

Previously a fatal affliction, WD is now treatable through chelation therapy(primarily penicillamine and trientine)and/or reducing intestinal copper absorption using zinc. All therapies can cause transient neurological deterioration in about 10% of individuals, recovery from which is often incomplete.^21^ Other rare side‐effects include hypersensitivity reactions(particularly with penicillamine), drug‐induced cytopenias^22^ and late development of nephrotic syndrome and drug‐induced lupus.^22^

### Inborn Errors of Metabolism

Inborn errors of metabolism (IEM) frequently manifest with complex neurological syndromes associated with liver involvement. Though individually rare, as a whole their cumulative frequency may exceed 1 in 1000, especially in ethic groups where consanguineous marriage is commonplace.^23^ Clinical features are highly heterogeneous, depending largely on residual enzyme/metabolic pathway function. This, combined with the sheer number of individual disorders (>1000) makes detailing specifics about individual conditions of limited utility in clinical practice.^24^ Saudubray et al. in 2019 proposed a simplified IEM classification system, grouping disorders according to the size of the affected molecule(“small” or “complex”), the nature of the defect (“accumulation” and “deficiency”) and the effects on energy production.^25^ This “clinically friendly” approach forms the basis of our discussion of IEMs. We also highlight red‐flags which should arouse suspicion, and detail an approach to testing (see Tables 2 and 3).

**TABLE 2:** Suggested screening for inborn errors of metabolism (IEM) according to clinical suspicion

The clinical similarities between many IEM makes it appropriate to cast a wide net as part of initial screening*.^26^ The first step should involve a review of newborn dried blood spot results (screened disorders vary among health systems). Thereafter, appropriate tests, depending on clinical suspicion, may include:
*Suspected small molecule “intoxication” disorder*
Serum ammonia level (may be normal outside of crises, especially in partial deficiency).
Urine organic acid analysis (detection of organic acidemias and some fatty acid oxidation defects)
Plasma and urine amino acids
Serum lactate
*Suspected small molecule “energy deficit” disorder*
Serum ketones and glucose (often both low during acute FAOD presentations)
Serum free fatty acids (FAOD)
Plasma acylcarnitine profile and serum free and total carnitines (low in disorders of carnitine transport)
Serum lactate:pyruvate ratio taken 1 hour after feeding (mitochondrial respiratory chain defects)^27^
Cerebrospinal fluid lactate, pyruvate, amino acids and protein^27^
Serum thymidine (especially in MNGIE)^27^
mtDNA genetic testing
Tissue analysis may be necessary to seal the diagnosis^27^
*Suspected disorder of “complex molecule” metabolism*
Lysosomal enzyme testing (most lysosomal storage disorders)
Plasma oxysterols (Niemann Pick type C)
Serum very long chain fatty acid, pristanic acid and phytanic acid analyses (peroxisomal disorders)

* Many of these tests are optimally performed after an overnight fast.^26^ Testing should be interpreted by a metabolic specialist, as it is oftentimes the pattern of abnormality, rather than the absolute individual values, which suggest the diagnosis.^28^

IEM, Inborn errors of metabolism; FAOD, fatty acid oxidation defects; MNGIE, mitochondrial neurogastrointestinal encephalopathy; mtDNA, mitochondrial DNA.

**TABLE 3:** Red flags for possible inborn errors of metabolism (note: The majority of these disorders are inherited in an autosomal recessive fashion)^29^

History
Parental consanguinity
Autosomal recessive inheritance pattern
Encephalopathic crises+/− triggers (infection, surgery, medications eg, valproate)
Neonatal death of a sibling
Protein aversion/preferred vegetarianism in patient or family members
Unexplained post‐partum neurological symptoms (urea cycle disorder)^30^
Episodic neurological symptoms
History of neonatal jaundice
Multi‐system involvement
Examination
Dysmorphism
Axial hypotonia
Spasticity
Seizures
Developmental delay
Hearing loss
Vision impairment
Stroke‐like episodes

#### Small Molecule “Accumulation” Disorders

This group includes the organic acidemias, urea cycle disorders and amino acid catabolism defects.^31^, ^32^ Most do not interfere with pre‐natal development, and present after a period (from hours to years) of apparent post‐natal normality, classically with acute, intermittent or progressive encephalopathy sometimes triggered by stressors such as dehydration, protein load, surgery, infection, fasting, or certain drugs.^25^ Features may include hypotonia, tremor, respiratory distress, vomiting, lethargy and dehydration, progressing if untreated to seizures, coma and death. Sepsis is often suspected, and a history of death of siblings, potentially mis‐attributed to infection, heart failure or intra‐ventricular hemorrhage may be present.^31^ Many of these disorders are treatable.

Partial enzyme deficiencies giving variant presentations in late childhood and adulthood are increasingly described, often manifesting with progressive motoric, psychiatric and behavioral syndromes (sometimes mis‐diagnosed as cerebral palsy).^30^ The most common MD observed are ataxia and dystonia.

Within this group, *glutaric aciduria type 1* is most commonly associated with MD, but liver involvement is rarely prominent. Another noteworthy condition is *ornithine transcarbamylase deficiency (OTCD)*, the most common urea cycle disorder. This X‐linked disorder usually presents in childhood, but adult presentations are increasingly recognized (eg, in males with partial deficiencies, or heterozygous females) and are an important differential diagnosis of adult hyperammonemic encephalopathy. Acute(sometimes recurrent) liver failure, is well recognized in OTCD, as are more minor degrees of liver dysfunction.^33^ Early measurement of serum ammonia, amino acid profile and urinary orotic and organic acids helps to seal the diagnosis.

#### Complex Molecule “Accumulation” Disorders

This group comprises probably the best recognized/defined IEMs, the lysosomal storage disorders (LSD), as well as rarer large molecule metabolism disorder eg, glycogenosis and neural lipid storage disorders.^25^, ^34^ As with their small molecule counterparts, they are generally devoid of antenatal manifestations but can present with progressive neurodegeneration during childhood or adult life, alongside variable “storage signs” eg, hepatomegaly, and other disease specific multi‐organ involvement. As with small molecule “accumulation” defects, some are (at least partially) treatable. An overview of some of these disorders is provided below:

*Gaucher disease*(GD), the most common LSD, is caused by recessively inherited mutations in the *GBA1* gene encoding the lysosomal enzyme, glucocerebrosidase. It results in accumulation of substrate‐laden macrophages (Gaucher cells) in the liver, spleen, bone marrow and other organs, producing the characteristic multi‐system manifestations.^35^

Systemic features, common to all sub‐types, comprise hepatosplenomegaly, bone involvement (stunted growth, painful bone crises) and cytopenias (most commonly thrombocytopenia).^35^ Liver enlargement is usually detected incidentally on physical examination or imaging, but only rarely associated with significant hepatic injury or fibrosis. Focal lesions‐usually benign Gaucheromas‐ are common, but GD patients have an increased risk of hepatocellular carcinoma, therefore this important differential diagnosis must always be kept in mind.^35^, ^36^

Type 1 GD, the most prevalent subtype, is usually diagnosed between 10 and 20 years of age, but may remain asymptomatic throughout life. It was previously considered free of neurological symptoms,^35^ but is now recognized to confer significantly increased risk of Parkinson's disease (often with more severe cognitive and non‐motor disease progression).^37^ Types 2 and 3 are earlier in onset. They may manifest pre‐natally as foetal hydrops, arthrogryposis and dermatologic abnormalities, or post‐natally as failure to thrive alongside neurological features such as bulbar dysfunction, spasticity and horizontal oculomotor abnormalities. Movement disorders, including progressive myoclonus epilepsy, ataxia and dystonia (dystonic opisthotonus, cervico‐facial dystonia or paroxysmal dystonic events) are frequently encountered.^38^, ^39^

Diagnosis is established by demonstrating deficient glucocerebrosidase activity in leukocytes or cultured fibroblast, and genetic analysis. Treatment is effective for the systemic, but not for neurological manifestations.^40^

*Niemann‐Pick disease type C* (NPC) is a progressive neuro‐visceral disorder caused by bi‐allelic mutations in either the *NPC1* (95% of cases) or *NPC2* genes, resulting in lysosomal accumulation of unesterified cholesterol and other lipids. It manifests along a spectrum ranging from a severe, rapidly progressive neonatal illness to slowly progressive late‐onset neurodegeneration.^41^

Early life presentations are often dominated by systemic manifestations‐liver disease with cholestatic jaundice, hepatosplenomegaly and occasionally, acute liver failure.^42^

In adolescent/adult‐onset cases, neuropsychiatric features are usually prominent.^43^ The combination of progressive ataxia, cognitive decline, vertical supra‐nuclear gaze palsy and psychiatric features is particularly suggestive.^44^ Dystonia (particularly affecting the limbs and/or face), seizures, deafness and gelastic cataplexy are other important features.^44^ Myoclonus, parkinsonism and chorea are also described.^42^, ^45^ All patients with NPC eventually become demented.^46^ Visceral manifestations may be subtle, and hepatomegaly may go unrecognized if not specifically sought.

Analysis of plasma oxysterols is the first step in diagnostic evaluation. Detection of pathogenic mutations in NPC1 and NPC2 genes confirms the diagnosis. Filipin staining of cultured fibroblasts is no longer recommended for initial screening.^42^

Neuropsychiatric manifestations progress with variable speed. Conversely, hepatic manifestations tend to remain stable or regress.^45^, ^47^ Miglustat may slow disease progression.^42^, ^43^

*GM1 gangliosidosis*, a rare disorder of reduced beta‐galactosidase activity, primarily manifests in infancy (type 1) or childhood (type 2) with coarse facial features, dysostosis, hepatosplenomegaly, ataxia, seizures, macular “cherry red” spot and progressive neurocognitive decline. Milder adult onset presentations (type 3) may be diagnostically challenging due to the absence of “typical” skeletal and neurovisceral findings.^48^, ^49^ Generalized dystonia with prominent perioral/facial involvement is almost universal, and may be accompanied by parkinsonism.^49^, ^50^ MRI brain is generally abnormal, showing bilateral putaminal T2‐weighted hyperintensities.^49^, ^50^ Diagnosis is established through beta‐galactosidase enzyme analysis or genetic testing of the *GLB1* gene.

The majority of *mucopoysaccharidoses(MPS)* have neurological involvement as a core feature.^34^ Dystonia, typically generalized with prominent facial/oromandibular involvement (sometimes producing characteristic “grimacing”) is the most typical MD, though ataxia and parkinsonism can also be seen.^50^ Hepatomegaly is a core disease feature, though despite its large size, hepatic dysfunction is not universal.^51^ Clinical pointers to MPS include coarse facial features, skeletal deformities, recurrent infections and hernias.

#### Small and Complex Molecule “Deficiency” Disorders

These disorders, which impair metabolism of critical small (eg, amino acids, fatty acids, metals) or large (eg, peroxisomal disorders) molecules distal to the defective metabolic process frequently impair neurodevelopment and often present at birth. Severe multi‐system anomalies with complex neurological phenotypes are frequent.

*Peroxisomal disorders* generally encompass varying degrees of liver dysfunction, neurological decline, retinopathy, renal involvement and sensorineural hearing loss.^52^ Foetal dysmorphism (particularly craniofacial) is common.^31^, ^52^ Neurological involvement range from infantile onset, rapidly progressive and lethal phenotypes through to late‐onset disease or even isolated hearing or visual problems.^52^ Brain imaging characteristically shows white matter signal abnormality, accompanied in severe cases by malformations of cortical development, callosal dysgenesis and brain atrophy. Cerebellar ataxia and dystonia are commonly observed MD, though tremor may be prominent in patients with alpha‐methylacyl‐CoA racemase (AMACR) deficiency.^52^

The nature and severity of liver involvement varies among individual disorders, but often comprises hepatomegaly alongside steatosis and/or steatohepatitis, occasionally progressing to liver failure.^53^ Elevated serum very long chain fatty acids(VLCFA) suggest the diagnosis, which is confirmed through genetic testing.

### Coeliac Disease

Coeliac disease (CD) is a multi‐system autoimmune disorder driven by dietary gluten ingestion in genetically susceptible individuals.^54^ It affects roughly 1% of the population, typically manifesting with symptomatic enteropathy. Extra‐intestinal manifestations‐dermatitis herpetiformis, arthritis, liver involvement and neurologic manifestations‐are also common.^54^

The cerebellum is particularly vulnerable to CD‐related injury,^55^ and MD in CD largely stem from cerebellar dysfunction.^56^ Gait ataxia is most common, though numerous “ataxia‐plus” syndromes, often comprising cortical myoclonus either as tremor or progressive myoclonic ataxia, have been described.^57^, ^58^, ^59^ Loss of cerebellar inhibition of cortical sensorimotor excitability is the likely driver of myoclonus.^60^ Another peculiar phenotype is stimulus sensitive/action‐induced foot myoclonus, which has frequently been associated with CD.^61^ These “cerebellar‐driven” phenotypes respond poorly, if at all, to gluten‐free diet or immunotherapy.

Chorea is the other major CD‐related MD. Almost all cases have been women presenting with sub‐acute generalized chorea in later life, though florid presentations and onset in young age are reported.^62^, ^63^ CD‐related chorea tends to respond well to gluten‐free diet.^62^, ^63^

Liver involvement occurs in up to 40% of adults and 60% of children with CD,^64^ and patients have 6 to 8‐fold greater risk of chronic liver disease as compared to the general population. Manifestations range from asymptomatic transaminitis (the most common presentation) to rapidly progressive liver failure.^64^ Most individuals with coeliac hepatitis show no outward signs of liver derangement, the disorder being picked up only through routine liver function testing.^64^

Biochemical and histologic markers of liver injury should improve within 6–12 months of institution of a gluten‐free diet—this confirms the diagnosis. Lack of improvement suggests co‐existence of another pathology (particularly autoimmune hepatobiliary disease and/or autoimmune hepatitis), established liver injury and fibrosis or a disease complication eg, lymphoma.^64^

Features which should make clinicians consider a diagnosis other than coeliac hepatitis include^64^:

1. Transaminase elevation >5xULN
2. Persistent transaminitis despite 6–12 months of gluten‐free diet
3. Hyperbilirubinemia

### Aceruloplasminemia

Aceruloplasminemia is a late‐onset autosomal recessive disorder combining systemic and brain iron overload, caused by mutations in the ceruloplasmin (*CP*) gene.^65^ Most cases have been reported from Japan.

Neurological symptom onset is generally between 40–60 years of age, though systemic features including diabetes mellitus and microcytic anemia are usually present for years beforehand.^65^ Symptoms include progressive cerebellar dysfunction, cognitive decline and choreodystonic movements of the cranio‐facial region.^65^, ^66^, ^67^ Caucasian patients more frequently develop parkinsonism compared to their Japanese counterparts, and are more likely to exhibit cognitive and/or psychiatric symptoms at disease outset.^65^, ^66^, ^67^ Retinopathy is common in Japanese patients, but is rarely symptomatic.^66^

Hepatic iron overload is almost universal, though symptomatic liver dysfunction and progression to cirrhosis are rarely reported.^66^, ^68^ Some cases show mild to moderate fibrosis on liver biopsy.^69^

Biochemical pointers to the diagnosis include reduced serum ceruloplasmin alongside elevated serum ferritin. Brain MRI usually demonstrates iron deposition in the basal ganglia, thalami and dentate nuclei. Definitive diagnosis requires identification of bi‐allelic pathogenic *CP* variants. Iron chelation is effective in reducing systemic iron overload, but its effectiveness for neurological disease remains dubious, and its administration not without potential side‐effects eg, cytopenias^66^; phlebotomy and fresh frozen plasma are also sometimes employed.

Symptomatic aceruloplasminemia has also recently been described among heterozygous *CP* mutation carriers. In these patients, neurological symptoms (particularly MD) predominate, systemic manifestations are rare, serum ferritin levels often normal and ceruloplasmin levels generally mildly below the lower limit of normal.^70^

### Ataxia Telangiectasia

Ataxia telangiectasia (AT) is a phenotypically diverse, multisystem autosomal recessive disorder characterized by progressive neurodegeneration, immunodeficiency and predisposition to malignancy.

The clinical features extend far beyond its double‐barreled eponym, and are influenced largely by residual ATM kinase activity rather than genotype.^71^ Most affected children manifest in a “classic” fashion, with progressive gait imbalance, falls and clumsiness first becoming evident around 12–18 months of age. They often have a severe disease course with progressive gait imbalance, loss of ambulation and eventual wheelchair dependency. Their childhood is often marred by recurrent sino‐pulmonary infections, stunted growth, and appearance of other MDs including dystonia, chorea, tremor and myoclonus.^71^ Most succumb to infection or malignancy within the first 3 decades of life.^72^

Up to 1/3 of affected individuals present later in life with milder “variant” AT. The dominant MD in such cases is often not ataxia, but rather dystonia (especially cervical, axial or myoclonus‐dystonia phenotypes) or chorea.^71^, ^72^ Most of these individuals develop ataxia over time, culminating in complex MD phenotypes.^71^

Liver disease develops in most patients with AT.^73^ Prevalence of steatosis and fibrosis correlate with disease duration, and most adults will exhibit abnormalities of liver function testing.^73^, ^74^ Due to the heightened risk for malignancy, hepatocellular carcinoma should also be considered in those with abnormal liver function, or hepatic imaging.

Clinical (cutaneous and conjunctival telangiectasias, oculomotor apraxia) and biochemical (elevated alpha‐fetoprotein) features may suggest the diagnosis,^71^ which is confirmed by genetic analysis of the *ATM* gene.

### Neuroacanthocytosis

Neuroacanthocytic disorders are characterized by progressive neurodegeneration in association with morphologically abnormal erythrocytes bearing thorn‐shaped surface projections (acanthocytes).^75^ The two principal disorders are autosomal recessive chorea acanthocytosis(caused by *VPS13A* gene mutations) and X‐linked McLeod syndrome(due to *XK* gene mutations, resulting in absence of the Kx blood group antigen).

Chorea acanthocytosis generally manifests in the 3rd–4th decade of life, whereas McLeod syndrome begins later(4th–5th decade). In both, MD are prominent, taking the form of generalized chorea, ataxia and marked perioral choreodystonic movements (particularly in chorea‐acanthocytosis) often producing early and prominent feeding problems. Stereotyped sudden lapses in axial and leg tone producing both head drops and a peculiar “rubber man” gait, may become evident later in the disease course.^76^ Secondary tics, parkinsonism, seizures, cognitive and neuropsychiatric abnormalities may also be evident.^75^ Axonal peripheral neuropathy and myopathy producing distal amyotrophy is common. The majority of patients with McLeod syndrome develop a cardiomyopathy.^75^ Serum CK (a cheap and simple test) is generally high, and LDH and liver enzymes often elevated. Hepatomegaly is seen especially in McLeod syndrome, but progression to cirrhosis or liver failure is not described.^75^, ^77^ Acanthocytes are often evident on blood film, though identification may require repeated testing. Some cases never exhibit acanthocytes, hence molecular genetic analysis is the preferred method of diagnosis. MRI brain generally shows striking caudate atrophy. Kell antigen testing is useful if considering McLeod syndrome.

Importantly, acanthocytes, or erythrocytes with similar morphological features (echniocytes, spur cells) can occur secondary to liver failure of any cause, potentially causing diagnostic confusion.

## Movement Disorders Occurring Secondary to Loss of Hepatic Detoxification Functions

One of the central roles played by the liver is that of detoxification, both of endogenous by‐products of metabolism and of exogenous substances. When these systems fail, neurotoxic compounds, such as ammonia, may accumulate in the bloodstream, cross the blood–brain‐barrier and producing neurological disturbances. This section largely encompasses two syndromes, one acute (hepatic encephalopathy) and one subacute/chronic(acquired hepatocerebral degeneration), which can be differentiated on the basis of history, clinical examination, neuroimaging and biochemical parameters.

### Acquired Hepatocerebral Degeneration

Acquired hepatocerebral degeneration (AHD) is a poly‐symptomatic neurobehavioral syndrome occurring in the setting of advanced liver disease.^78^ Its prevalence depends on the population studied, ranging from about 2% of unselected cirrhotic patient to >20% of people with end‐stage liver disease.^79^ AHD can result from any liver affectation,^80^, ^81^ though a critical factor appears to be the development of porto‐systemic shunting.^80^ Most affected patients are between 40 and 70 years of age,^79^, ^80^, ^81^ but children are not immune.^82^

The most common phenotype of AHD is that of atypical parkinsonism, which in contrast to idiopathic Parkinson's disease (PD), is bilateral, symmetrical, and accompanied by postural(rather than rest) tremor and early ataxic gait impairment and falls.^80^ Other phenotypes are described, including an “ataxia‐plus” disorder (with coexisting dystonia, seizures or tremor) and a neuropsychiatric syndrome.^81^ Onset is generally sub‐acute over several weeks to months, often coincident with worsening of liver disease and reaches maximal severity after an average of 7 months.^80^ Cognitive involvement is common, and patients are frequently apathetic and bradyphrenic, though language and praxis is generally preserved.^79^, ^80^, ^81^ Response to levodopa is variable.^81^

Other MD may be apparent. Up to half exhibit focal dystonia, most commonly involving the face (blepharospasm and/or oromandibular dystonia),^80^ which may resemble tardive orobuccolingual movements.^79^, ^83^ Dysarthria and dysphagia are frequent. Leg tremor is distinctly unusual.^80^

All patients exhibit bilateral pallidal T1‐hyperintensities on MRI, reflecting pallidal manganese deposition which is critical to AHD pathogenesis.^80^, ^81^, ^84^ These changes are identical to those observed in other forms of manganism, including from occupational exposure, total parenteral nutrition and illicit drug use.^80^, ^85^ Radiologic findings alone cannot be relied upon to make the diagnosis, as these are present in 75%–100% of cirrhotic individuals, regardless of the presence of neurological symptoms.^86^, ^87^ Serum manganese levels are always elevated, though the degree of elevation does not correlate with neuroimaging abnormalities.^80^, ^85^ Liver transplantation often leads to normalization of serum manganese levels and brain MRI changes, but the effects on the clinical syndrome may be minimal.^81^ If recovery ensues, it is generally slow, though lazaroid improvements within hours of surgery have been reported.^79^, ^83^, ^88^

### Hepatic Encephalopathy

Along with coagulopathy and jaundice, hepatic encephalopathy(HE) is a cardinal indicator of critical failure of hepatic synthetic and detoxifying functions. It heralds a significant change in disease course‐severe hepatic encephalopathy carries a 50% mortality at 1 year.^89^

HE comprises two principal components: a) a disorder of vigilance and arousal, and b) neuromuscular excitability. Conscious alterations exist along a spectrum. Early symptoms include poor attention, sleep–wake cycle disturbances and anxiety. If unchecked, these progress to personality change, dis‐inhibition, apathy, disorientation, memory impairment and eventually coma and obtundation.^90^, ^91^

Negative myoclonus is the other hallmark feature of HE, often manifesting as the flapping tremor of asterixis. This is easily appreciated in the upper limbs, though is often apparent in other regions if tested.^92^ Importantly, asterixis is not pathognomonic of liver dysfunction, and can be observed in other, often metabolic, derangements.

From a pathomechanistic and management perspective, HE should be considered according to the acuity of its development. Most HE is of the “chronic” type, occurring as it were in patients with longstanding cirrhosis who experience a slow progressive decline in liver function, leading to portosystemic shunting of toxic substances, which at some point reaches a critical threshold such that neurological impairment becomes manifest. HE in this population is frequently triggered by extraneous factors eg, sepsis, electrolyte disturbance, constipation or intestinal hemorrhage. In contrast, HE occurring in patients with acute or fulminant liver failure, defined as rapid (<26 weeks) development of encephalopathy and hepatic synthetic dysfunction (INR≥1.5) in a patient without pre‐existing cirrhosis or chronic liver disease, is more problematic. Rapid osmotic shifts can produce significant cerebral oedema, increased intracranial pressure and the threat of brain herniation.^93^

In “chronic” HE, serum ammonia levels are often elevated, but there is little correlation with the presence and/or severity of the neurocognitive syndrome.^94^ In contrast, ammonia levels do predict outcomes in acute hepatic failure, where venous ammonia in excess of 150 μmol/L is an independent predictors of increased intracranial pressure, cerebral herniation and death.^94^, ^95^

Treatment of HE aims to reduce systemic ammonia load. Intestinal ammonia production is reduced through administration of non‐absorbable disaccharides, sometimes supplemented by oral antimicrobials.^93^, ^96^ This should occur concurrently with reversing any triggers of hepatic decompensation eg, infection, gastrointestinal bleeding or drug/toxin administration, and consideration of liver transplantation. In those with “acute” HE, clinical and neuroradiologic signs of increased intracranial pressure should be carefully sought, and the patient aggressively managed in a neurocritical care environment.^93^

HE and AHD are commonly conflated in the literature, but they are distinct entities with different pathomechanisms, responses to treatment and clinical courses. Differentiating between them is rarely cumbersome, though both may coexist at certain points in time. The following pointers are useful to keep in mind:

- Altered level of consciousness, a core feature of HE, is absent in AHD.
- AHD generally assumes an akinetic‐rigid phenotype, whereas abnormal movements in HE are usually hyperkinetic(particularly myoclonic).
- Clinical deficits of HE often respond to ammonia‐lowering therapies. Those of AHD do not.

## Combined Movement Disorders and Hepatic Disease Secondary to Toxic/Iatrogenic Causes

Combined hepatic and neurological syndromes secondary to intake of exogenous substances, either recreationally or as prescription medication, ranks among the most common causes of MD and hepatic disease. Below is a summary of the most common scenarios to consider:

### Alcohol‐Related Disease

Alcohol produces nefarious effects principally on the central nervous system and liver, and tops the list of toxic substances combining MD and liver disease. Movement disorders are a common symptom of alcohol‐related CNS disease, and primarily exhibit one of two phenotypes:

#### Ataxia

Ataxia is the principal MD encountered following alcohol ingestion, and can occur in any number of clinical scenarios:

1.Acute Intoxication

The features of acute alcohol intoxication are highly variable. They depend on the quantity ingested, the period of time over which ingestion occurred, body habitus, tolerance and co‐ingestion of other toxic substances.^97^ Neurologic symptoms follow a dose‐dependent pattern, beginning with increased talkativeness and relaxation, followed by slurred speech, impaired judgment, lack of coordination and ataxia.^97^ Examination during moderate intoxication often reveals gait ataxia as well as saccadic ocular pursuits and other cerebellar eye signs.^98^

2.Cerebellar degeneration

Alcoholic cerebellar degeneration is the most common acquired toxic ataxia, affecting up to 50% of long‐term alcohol abusers.^99^ The syndrome is one of pronounced gait and stance ataxia with relative absence of upper limb, speech or oculomotor deficits. The ataxia often begins subacutely after many years of chronic alcoholism and is frequently progressive. Brain imaging typically shows cerebellar atrophy with preferential involvement of the vermis.^100^ Early theories favored nutritional deficiencies and/or direct cellular toxicity as an aetiopathologic explanation, though recent evidence suggests immune mechanisms may be at play.^100^

1. Nutritional syndromes

Multifactorial deficiencies in B‐vitamins, particularly thiamine, are common in chronic alcoholics, and can impair diencephalic, brainstem and cerebellar functions.^101^ Wernicke's encephalopathy (WE) is a largely reversible syndrome manifesting with varying degrees of confusion, ataxia and ophthalmoplegia.^98^ If unchecked, Korsakoff syndrome may ensue, the most striking feature of which is marked, especially anterograde amnesia.^98^, ^101^

### Tremor

Tremor occurs both in the setting of chronic alcoholism, and of alcohol withdrawal. Over half of chronic alcoholics experience tremor, most commonly large‐amplitude postural hand tremor.^102^ This is rarely disabling, and its severity decreases with continued abstinence.^92^ Other tremors have been described in chronic alcoholics, including position‐dependent upper‐limb rest tremor resembling parkinsonian tremor, and 3 Hz leg rest tremor, which is best appreciated either in the standing position with the knees bent, causing a bobbing motion, or when lying supine with hips and knees flexed to 90 degrees, as a kicking movement.^92^ Tremor also emerges following intentional or unintentional abrupt cessation of chronic, heavy drinking, alongside other symptoms of alcohol withdrawal such as hallucinations (visual, tactile, auditory), autonomic activation and seizures.^103^

Other MD occurring in the setting of alcohol withdrawal include transient parkinsonism, myoclonus, dystonia and a variety of choreiform movements.^104^, ^105^, ^106^, ^107^

The manifestations of alcoholic liver disease are vast. Most problem drinkers will develop alcoholic steatosis, which is generally reversible upon stopping alcohol consumption.^108^, ^109^ With continued drinking, progression to steatohepatitis and eventually fibrosis and cirrhosis occurs. However, only 10%–20% of people with alcoholic steatosis who continue to drink will progress to fibrosis, indicating a significant role for other modifiers in determining disease expression‐these include age, sex, race, comorbid conditions such as diabetes and obesity, and genetics.^108^, ^110^

Importantly, a number of MD, particularly essential tremor and myoclonus‐dystonia may be alcohol‐responsive, resulting in over‐indulgence in order to mitigate disability.

Thiamine supplementation should be given for all cases of suspected or confirmed alcohol‐related neurological disease.

### Illicit Drug Use

The association of MD alongside hepatic disease should always lead to consideration of illicit drug use. At‐risk behaviors in drug abusers place them at heightened risk for liver disease, not only because of co‐infection with hepatitis viruses, but also because of a propensity for heavy alcohol consumption. Prevalence of hepatitis C in intravenous drug users is up to 90%,^111^ and over 50% meet criteria for harmful/hazardous drinking.^112^ Up to 25% of injection drug users will have cirrhosis.^113^, ^114^

Drug‐induced MD either result from the pharmacologic properties of the ingested substance or from adulterants.^115^ Common toxindromes are detailed below:

*Cocaine* blocks the pre‐synaptic re‐uptake of dopamine and other catecholamines, with euphoric and motoric consequences. Transient orobuccal and limb chorea, known as “crack dancing”, is a dramatic phenotypic manifestation which can persist for several days.^115^ Motor and verbal tics are also common. Chronic abuse can lead to down‐regulation of dopaminergic transmission, producing parkinsonism during periods of abstinence.^116^ Interestingly, prior neuroleptic use may be a risk factor for the development of cocaine‐related MD; conversely, cocaine may precipitate acute dystonic reactions in neuroleptic users.^115^, ^117^, ^118^

*Amphetamine* and *methamphetamine* act similarly to cocaine, producing euphoria, sympathetic arousal, chorea, tremor, ataxia and seizures.^115^, ^119^ Delusions of formication are a common neuropsychiatric feature of chronic use, as is punding.^119^

*Ephedrone*, synthesized from pseudoephedrine using potassium permanganate as an oxidant, is predominantly encountered in former Soviet states. Some users have developed striking subacute‐onset akinetic‐rigid parkinsonism due to secondary manganism‐the likely culprit being the oxidizing agent.^115^, ^120^

### Medications Commonly Administered for Liver Diseases

Treatment of liver diseases varies according to etiology, severity and patient comorbidities. Numerous medications may be employed and these should always be considered as potentially causative of observed MD.

Two particularly important examples are given below:

#### Calcineurin Inhibitors

Liver transplant is the treatment of choice for chronic decompensated liver failure and fulminant hepatic failure of any etiology. Improvements in immunosuppressive regimes in recent decades have significantly improved patient and graft survival.^121^ This however comes with complications such as malignancies, opportunistic infections and organ toxicities.^121^ Neurotoxicity is particularly encountered with the calcineurin inhibitors(CNI), ciclosporin and tacrolimus.

Neurotoxicity affects most CNI‐treated patients. Mild manifestations such as tremor affect up to 80% of CNI (especially tacrolimus)‐treated patients. This is generally a symmetrical rest and action tremor of the upper limbs, though facial and lower limb muscles may be involved.^122^ Other rarer manifestations include parkinsonism, opisthotonus and severe rigidity.^123^ Severe toxicity may progress to stupor and coma.^124^ Neurological effects are not strictly dose‐dependent, and can occur with normal drug trough levels. MRI brain may demonstrate features supportive of CNI‐neurotoxicity such as PRES or cortical hyperintensity, particularly in the cingulate or occipital regions.^125^ Management involves dose reduction, and therefore requires close cooperation with the transplant team.

#### Metronidazole

Metronidazole is occasionally in the management of hepatic encephalopathy, or for treatment of intercurrent infections in patients with liver disease.^126^ Patients receiving metronidazole may develop a syndrome characterized by ataxia, cerebellar dysarthria, neuropathy and occasionally, encephalopathy.^127^, ^128^ Toxicity can result either from high‐dose or cumulative low dose exposure. Importantly, hepatic dysfunction may predispose to this unusual complication.^128^ MRI brain classically shows prominent, reversible, T2‐weighted and FLAIR signal hyperintensity in the dentate nuclei.^127^, ^128^

### Medications Commonly Administered for Movement Disorders

The principal drugs with hepatotoxic potential prescribed in MD practice are valproate and tolcapone.^129^ *Valproate* is employed in a variety of clinical scenarios for mood stabilization, seizure control and relief of myoclonus. It can produce a number of hepatic syndromes, including:

‐ Symptomatic isolated hyperammonemia with minimal or no evidence of liver dysfunction, usually manifesting within weeks of treatment initiation or dose escalation with progressive obtundation and confusion, which is reversible upon stopping treatment.^130^

‐ Acute hepatocellular injury, with significant transaminitis, hyperbilirubinemia and hepatic synthetic dysfunction occasionally progressing to liver failure.

‐ Reye‐like syndrome in children receiving valproate following viral infection.

*Tolcapone* is a catechol‐O‐methyltransferase inhibitor used as an adjunctive therapy in the management of PD. In up to 5% of patients, it results in significant transaminase elevation. Though usually self‐limiting, some people have experienced progressive, sometimes lethal hepatocellular injury.^131^

Intake of medications for other ailments (Table 4) as well as herbal and/or traditional medications should also be considered, as they can have significant hepatotoxic potential.

**TABLE 4:** Examples of prescribed medications which may cause derangements in liver function testing (along with usual pattern of liver injury)

Predominantly hepatocellular	Mixed	Predominantly cholestatic
‐NSAIDs ‐Isoniazid ‐Antifungals ‐Statins ‐Allopurinol ‐Amiodarone ‐Paracetamol ‐Valproate	‐Sulfonamides ‐HAART(esp. non‐nucleoside analog reverse transcriptase inhibitors) ‐ Antibiotics(esp. clavulanic acid containing compounds) ‐Phenytoin ‐Carbamazepine ‐Phenobarbital	‐Rifampicin ‐Antibiotics (esp. clavulanic acid containing compounds, penicillinase‐resistant penicillins and macrolides) ‐Carbamazepine ‐Tricyclic antidepressants ‐oral contraceptives ‐Anabolic steroids

HAART, highly active anti‐retroviral therapy; NSAIDs, non‐steroidal anti‐inflammatory medications.

## Conclusion

The myriad of clinically and pathophysiologically distinct conditions combining MD and liver disease can, at first, seem bewildering. Many of the disorders discussed herein (particularly inherited causes) are vanishingly rare, their systemic manifestations non‐specific and their MD phenotypes incompletely characterized.^29^ Moreover, partial enzyme deficiencies producing variant presentations in adulthood are increasingly being recognized. One must also remember that combined MD and liver symptomatology can be the product of simple happenstance, due for example to two independent organ pathologies, diffuse metastatic or inflammatory disease.

Nevertheless, a systematic approach considering age of onset, MD phenomenology, developmental and family history and other neurological/systemic features can effectively guide diagnostic testing (Fig. 1). For example, early age at onset, complex neurological phenotypes and other “red flags” suggest inherited IEMs; conversely, stigmata of chronic liver disease or a history of alcohol or substance misuse may rather suggest acquired disorders stemming from critical failure of hepatic detoxifying functions. Wilson's disease should always be excluded. Disentangling the syndrome may require exhaustive testing and multidisciplinary collaborations, but given the treatable nature of many of these conditions, is certainly worthwhile.

## Author Roles

(1) Research project: A. Conception, B. Organization, C. Execution; (2) Statistical Analysis: A. Design, B. Execution, C. Review and Critique; (3) Manuscript Preparation: A. Writing of the first draft, B. Review and Critique.

EM: 1A, 1B, 1C, 2A, 2B, 2C, 3A

FB: 1A, 1B, 1C, 2A, 2B, 2C, 3A

FM: 1B, 1C, 2C, 3B

AL: 1B, 1C, 2C, 3B

PC: 1B, 1C, 2C, 3B

KPB: 1B, 1C, 2C, 3B

## Disclosures

### Ethical Compliance Statement

We confirm that we have read the Journal's position on issues involved in ethical publication and affirm that this work is consistent with those guidelines. Patient consent and institutional review board approval were not required for this work.

### Funding Sources and Conflict of Interest

No specific funding was received for this work. The authors declare that there are no conflicts of interest relevant to this work.

### Financial Disclosures for the previous 12 months

EM is supported by the Edmond J. Safra Foundation and the National Institute for Health Research University College London Hospitals Biomedical Research Centre. FM is supported by the European Academy of Neurology (EAN) Research Fellowship 2020. KPB holds research grants from EU Horizon 2020 and has received honoraria to speak at meetings or to attend advisory boards from Ipsen, Cavion, Allergan, Teva Lundbeck and Bial pharmaceutical companies. He also receives royalties from Oxford University Press and a stipend for MDCP editorship. FB, AL and PC report no disclosures.

## Supporting information

**Table S1.** Inherited diseases with movement disorders and liver involvement.

**Table S2.** Acquired diseases with movement disorders and liver involvement.

**Supplementary material S3.** Methods.

## References

[1] Macpherson I, Nobes JH, Dow E, et al. Intelligent liver function testing: working smarter to improve patient outcomes in liver disease. J Appl Lab Med 2020;5:1090–1100. 10.1093/jalm/jfaa109. 32916711

[2] Lindenmeyer CC, McCullough AJ. The natural history of nonalcoholic fatty liver disease—An evolving view. Clin Liver Dis 2018;22:11–21. 10.1016/j.cld.2017.08.003. 29128051 PMC6130315

[3] Loomba R, Adams LA. Advances in non‐invasive assessment of hepatic fibrosis. Gut 2020;69:1343–1352. 10.1136/gutjnl-2018-317593. 32066623 PMC7945956

[4] Rosencrantz R, Schilsky M. Wilson disease: pathogenesis and clinical considerations in diagnosis and treatment. Semin Liver Dis 2011;31:245–259. 10.1055/s-0031-1286056. 21901655

[5] Ferenci P, Stremmel W, Członkowska A, et al. Age and sex but not ATP7B genotype effectively influence the clinical phenotype of Wilson disease. Hepatology 2019;69:1464–1476. 10.1002/hep.30280. 30232804

[6] Wallace DF, Dooley JS. ATP7B variant penetrance explains differences between genetic and clinical prevalence estimates for Wilson disease. Hum Genet 2020;139:1065–1075. 10.1007/s00439-020-02161-3. 32248359

[7] Boga S, Ala A, Schilsky ML. Hepatic features of Wilson disease. Handbook of Clinical Neurology; 2017:91–99. 10.1016/B978-0-444-63625-6.00009-4. 28433114

[8] Merle U, Schaefer M, Ferenci P, et al. Clinical presentation, diagnosis and long‐term outcome of Wilson's disease: a cohort study. Gut 2007;56:115–120. 10.1136/gut.2005.087262. 16709660 PMC1856673

[9] Dabrowska E, Jabłońska‐Kaszewska I, Oziebłowski A, et al. Acute haemolytic syndrome and liver failure as the first manifestations of Wilson's disease. Med Sci Monit 2001;7(Suppl 1):246–251. 12211729

[10] Korman JD, Volenberg I, Balko J, et al. Screening for Wilson disease in acute liver failure: a comparison of currently available diagnostic tests. Hepatology 2008;48:1167–1174. 10.1002/hep.22446. 18798336 PMC4881751

[11] Roberts E. A practice guideline on Wilson disease. Hepatology 2003;37:1475–1492. 10.1053/jhep.2003.50252. 12774027

[12] WILSON SAK. Progressive lenticular degeneration: a familial nervous disease associated with cirrhosis of the liver. Brain 1912;34:295–507. 10.1093/brain/34.4.295. 19634211

[13] Dusek P, Litwin T, Członkowska A. Neurologic impairment in Wilson disease. Ann Transl Med 2019;7:S64. 10.21037/atm.2019.02.43. 31179301 PMC6531649

[14] Członkowska A, Litwin T, Chabik G. Wilson disease. Handbook of Clinical Neurology 2017:101–119. 10.1016/B978-0-444-63625-6.00010-0. 28433096

[15] Hedera P. Wilson's disease: a master of disguise. Parkinsonism Relat Disord 2019;59:140–145. 10.1016/j.parkreldis.2019.02.016. 30797706

[16] Mulroy E, Balint B, Adams ME, et al. Animals in the brain. Mov Disord Clin Pract 2019;6:189–198. 10.1002/mdc3.12734. 30949548 PMC6417842

[17] Palumbo CS, Schilsky ML. Clinical practice guidelines in Wilson disease. Ann Transl Med 2019;7:S65. 10.21037/atm.2018.12.53. 31179302 PMC6531645

[18] Roberts EA, Schilsky ML. Diagnosis and treatment of Wilson disease: an update. Hepatology 2008;47:2089–2111. 10.1002/hep.22261. 18506894

[19] Perman JA, Werlin SL, Grand RJ, et al. Laboratory measures of copper metabolism in the differentiation of chronic active hepatitis and Wilson disease in children. J Pediatr 1979;94:564–568. 10.1016/S0022-3476(79)80011-6. 430291

[20] Stättermayer AF, Entenmann A, Gschwantler M, et al. The dilemma to diagnose Wilson disease by genetic testing alone. Eur J Clin Invest 2019;49:e13147. 10.1111/eci.13147. 31169307 PMC6772051

[21] Litwin T, Dzieżyc K, Karliński M, et al. Early neurological worsening in patients with Wilson's disease. J Neurol Sci 2015;355:162–167. 10.1016/j.jns.2015.06.010. 26071888

[22] Aggarwal A, Bhatt M. Advances in treatment of Wilson disease. Tremor Other Hyperkinet Mov 2018;8:525. 10.7916/D841881D. PMC5840318 29520330

[23] Sanderson S, Green A, Preece MA, et al. The incidence of inherited metabolic disorders in the west midlands, UK. Arch Dis Child 2006;91:896–899. 10.1136/adc.2005.091637. 16690699 PMC2082934

[24] Ferreira CR, van Karnebeek CDM, Vockley J, et al. A proposed nosology of inborn errors of metabolism. Genet Med 2019;21:102–106. 10.1038/s41436-018-0022-8. 29884839 PMC6286709

[25] Saudubray J, Mochel F, Lamari F, et al. Proposal for a simplified classification of IMD based on a pathophysiological approach: a practical guide for clinicians. J Inherit Metab Dis 2019;42:706–727. 10.1002/jimd.12086. 30883825

[26] Guerrero RB, Salazar D, Tanpaiboon P. Laboratory diagnostic approaches in metabolic disorders. Ann Transl Med 2018;6:470–470. 10.21037/atm.2018.11.05. 30740401 PMC6331366

[27] Molleston JP, Sokol RJ, Karnsakul W, et al. Evaluation of the child with suspected mitochondrial liver disease. J Pediatr Gastroenterol Nutr 2013;57:269–276. 10.1097/MPG.0b013e31829ef67a. 23783016 PMC3810178

[28] Yıldız Y, Sivri HS. Inborn errors of metabolism in the differential diagnosis of fatty liver disease. Turk J Gastroenterol 2020;31:3–16. 10.5152/tjg.2019.19367. 32009609 PMC7075690

[29] Ebrahimi‐Fakhari D, Van Karnebeek C, Münchau A. Movement disorders in treatable inborn errors of metabolism. Mov Disord 2019;34:598–613. 10.1002/mds.27568. 30557456

[30] Häberle J, Boddaert N, Burlina A, et al. Suggested guidelines for the diagnosis and management of urea cycle disorders. Orphanet J Rare Dis 2012;7:32. 10.1186/1750-1172-7-32. 22642880 PMC3488504

[31] Saudubray J‐M. Clinical approach to inborn errors of metabolism in Paediatrics. Inborn Metabolic Diseases. Berlin, Heidelberg, Germany: Springer Berlin Heidelberg; 2012:3–54. 10.1007/978-3-642-15720-2_1.

[32] Saudubray J‐M, Garcia‐Cazorla A. An overview of inborn errors of metabolism affecting the brain: from neurodevelopment to neurodegenerative disorders. Dialogues Clin Neurosci 2018;20:301–326. 10.31887/DCNS.2018.20.4/jmsaudubray. 30936770 PMC6436954

[33] Laemmle A, Gallagher RC, Keogh A, et al. Frequency and pathophysiology of acute liver failure in ornithine Transcarbamylase deficiency (OTCD). PLoS One 2016;11:e0153358. 10.1371/journal.pone.0153358. 27070778 PMC4829252

[34] Bellettato CM, Hubert L, Scarpa M, et al. Inborn errors of metabolism involving complex molecules. Pediatr Clin North Am 2018;65:353–373. 10.1016/j.pcl.2017.11.011. 29502918

[35] Stirnemann J, Belmatoug N, Camou F, et al. A review of Gaucher disease pathophysiology, clinical presentation and treatments. Int J Mol Sci 2017;18:441. 10.3390/ijms18020441. PMC5343975 28218669

[36] Adar T, Ilan Y, Elstein D, et al. Liver involvement in Gaucher disease—Review and clinical approach. Blood Cells Mol Dis 2018;68:66–73. 10.1016/j.bcmd.2016.10.001. 27842801

[37] Avenali M, Blandini F, Cerri S. Glucocerebrosidase defects as a major risk factor for Parkinson's disease. Front Aging Neurosci 2020;12:97. 10.3389/fnagi.2020.00097. 32372943 PMC7186450

[38] Kraoua I, Sedel F, Caillaud C, et al. A French experience of type 3 Gaucher disease: phenotypic diversity and neurological outcome of 10 patients. Brain Dev 2011;33:131–139. 10.1016/j.braindev.2010.02.005. 20307947

[39] Weiss K, Gonzalez AN, Lopez G, et al. The clinical management of type 2 Gaucher disease. Mol Genet Metab 2015;114:110–122. 10.1016/j.ymgme.2014.11.008. 25435509 PMC4312716

[40] Gary SE, Ryan E, Steward AM, et al. Recent advances in the diagnosis and management of Gaucher disease. Expert Rev Endocrinol Metab 2018;13:107–118. 10.1080/17446651.2018.1445524. 30058864 PMC6129380

[41] Newton J, Milstien S, Spiegel S. Niemann‐pick type C disease: the atypical sphingolipidosis. Adv Biol Regul 2018;70:82–88. 10.1016/j.jbior.2018.08.001. 30205942 PMC6327306

[42] Patterson MC, Clayton P, Gissen P, et al. Recommendations for the detection and diagnosis of Niemann‐pick disease type C. Neurol Clin Pract 2017;7:499–511. 10.1212/CPJ.0000000000000399. 29431164 PMC5800709

[43] Patterson MC, Hendriksz CJ, Walterfang M, et al. Recommendations for the diagnosis and management of Niemann–pick disease type C: an update. Mol Genet Metab 2012;106:330–344. 10.1016/j.ymgme.2012.03.012. 22572546

[44] Evans WRH, Hendriksz CJ. Niemann‐pick type C disease—The tip of the iceberg? A review of neuropsychiatric presentation, diagnosis and treatment. BJPsych Bull 2017;41:109–114. 10.1192/pb.bp.116.054072. 28400970 PMC5376728

[45] Sevin M, Lesca G, Baumann N, et al. The adult form of Niemann‐pick disease type C. Brain 2006;130:120–133. 10.1093/brain/awl260. 17003072

[46] Mulroy E, Jaunmuktane Z, Balint B, et al. Some new and unexpected Tauopathies in movement disorders. Mov Disord Clin Pract 2020;7:616–626. 10.1002/mdc3.12995. 32775506 PMC7396854

[47] Vanier MT. Niemann–Pick diseases. Handbook of Clinical Neurology 2013:1717–1721. 10.1016/B978-0-444-59565-2.00041-1. 23622394

[48] Goldman JE, Katz D, Rapin I, et al. Chronic GM1 gangliosidosis presenting as dystonia: I. clinical and pathological features. Ann Neurol 1981;9:465–475. 10.1002/ana.410090509. 6791574

[49] Yoshida K, Oshima A, Sakuraba H, et al. GM1 gangliosidosis in adults: clinical and molecular analysis of 16 Japanese patients. Ann Neurol 1992;31:328–332. 10.1002/ana.410310316. 1353343

[50] Muthane U, Chickabasaviah Y, Kaneski C, et al. Clinical features of adult G M1 gangliosidosis: report of three Indian patients and review of 40 cases. Mov Disord 2004;19:1334–1341. 10.1002/mds.20193. 15389993

[51] Krawiec P, Pac‐Kożuchowska E, Mełges B, et al. From hypertransaminasemia to mucopolysaccharidosis IIIA. Ital J Pediatr 2014;40:97. 10.1186/s13052-014-0097-z. 25439061 PMC4260237

[52] Tan AP, Gonçalves FG, Almehdar A, et al. Clinical and neuroimaging Spectrum of Peroxisomal disorders. Top Magn Reson Imaging 2018;27:241–257. 10.1097/RMR.0000000000000172. 30086110

[53] Baes M, Van Veldhoven PP. Hepatic dysfunction in peroxisomal disorders. Biochim Biophys Acta 1863;2016:956–970. 10.1016/j.bbamcr.2015.09.035. 26453805

[54] Lindfors K, Ciacci C, Kurppa K, et al. Coeliac disease. Nat Rev Dis Primers 2019;5:3. 10.1038/s41572-018-0054-z. 30631077

[55] Hadjivassiliou M, Aeschlimann P, Sanders DS, et al. Transglutaminase 6 antibodies in the diagnosis of gluten ataxia. Neurology 2013;80:1740–1745. 10.1212/WNL.0b013e3182919070. 23576621

[56] Rouvroye MD, Zis P, Van Dam A‐M, et al. The neuropathology of gluten‐related neurological disorders: a systematic review. Nutrients 2020;12:822. 10.3390/nu12030822. PMC7146117 32244870

[57] Bhatia KP, Brown P, Gregory R, et al. Progressive myoclonic ataxia associated with coeliac disease. Brain 1995;118:1087–1093. 10.1093/brain/118.5.1087. 7496772

[58] Bürk K, Farecki M‐L, Lamprecht G, et al. Neurological symptoms in patients with biopsy proven celiac disease. Mov Disord 2009;24:2358–2362. 10.1002/mds.22821. 19845007

[59] Fung VSC, Duggins A, Morris JGL, et al. Progressive myoclonic ataxia associated with celiac disease presenting as unilateral cortical tremor and dystonia. Mov Disord 2000;15:732–734. 10.1002/1531-8257(200007)15:4<732::AID-MDS1021>3.0.CO;2-J. 10928587

[60] Latorre A, Rocchi L, Magrinelli F, et al. Unravelling the enigma of cortical tremor and other forms of cortical myoclonus. Brain 2020;143:2653–2663. 10.1093/brain/awaa129. 32417917

[61] Jesús S, Latorre A, Vinuela A, et al. Stimulus sensitive foot myoclonus: a clue to coeliac disease. Mov Disord Clin Pract 2019;6:320–323. 10.1002/mdc3.12753. 31061841 PMC6476595

[62] Pereira AC, Edwards MJ, Buttery PC, et al. Choreic syndrome and coeliac disease: a hitherto unrecognised association. Mov Disord 2004;19:478–482. 10.1002/mds.10691. 15077250

[63] Lefter S, Corcoran L, McAuliffe E, et al. Coeliac disease presenting with chorea. Pract Neurol 2020;20:144–147. 10.1136/practneurol-2019-002396. 31780451

[64] Rubio‐Tapia A, Murray JA. Liver involvement in celiac disease. Minerva Med 2008;99:595–604. 19034257 PMC3941070

[65] Vroegindeweij LHP, van der Beek EH, Boon AJW, et al. Aceruloplasminemia presents as type 1 diabetes in non‐obese adults: a detailed case series. Diabet Med 2015;32:993–1000. 10.1111/dme.12712. 25661792

[66] Marchi G, Busti F, Zidanes AL, et al. Aceruloplasminemia: a severe neurodegenerative disorder deserving an early diagnosis. Front Neurosci 2019;13:1–8. 10.3389/fnins.2019.00325. 31024241 PMC6460567

[67] Miyajima H. Aceruloplasminemia, an iron metabolic disorder. Neuropathology 2003;23:345–350. 10.1046/j.1440-1789.2003.00521.x. 14719552

[68] Vila Cuenca M, Marchi G, Barqué A, et al. Genetic and clinical heterogeneity in thirteen new cases with Aceruloplasminemia. Atypical anemia as a clue for an early diagnosis. Int J Mol Sci 2020;21:2374. 10.3390/ijms21072374. PMC7178074 32235485

[69] Pelucchi S, Mariani R, Ravasi G, et al. Phenotypic heterogeneity in seven Italian cases of aceruloplasminemia. Parkinsonism Relat Disord 2018;51:36–42. 10.1016/j.parkreldis.2018.02.036. 29503155

[70] Borges MD, Albuquerque DM, Lanaro C, et al. Clinical relevance of heterozygosis for aceruloplasminemia. Am J Med Genet B Neuropsychiatr Genet 2019;180:266–271. 10.1002/ajmg.b.32723. 30901137

[71] Levy A, Lang AE. Ataxia‐telangiectasia: a review of movement disorders, clinical features, and genotype correlations. Mov Disord 2018;33:1238–1247. 10.1002/mds.27319. 29436738

[72] Smith LL, Conerly SL. Ataxia‐telangiectasia or Louis‐bar syndrome. J Am Acad Dermatol 1985;12:681–696. 10.1016/S0190-9622(85)70094-1. 2580869

[73] Donath H, Woelke S, Theis M, et al. Progressive liver disease in patients with ataxia telangiectasia. Front Pediatr 2019;7:458. 10.3389/fped.2019.00458. 31788461 PMC6856634

[74] Weiss B, Krauthammer A, Soudack M, et al. Liver disease in pediatric patients with ataxia telangiectasia. J Pediatr Gastroenterol Nutr 2016;62:550–555. 10.1097/MPG.0000000000001036. 26594831

[75] Rampoldi L, Danek A, Monaco AP. Clinical features and molecular bases of neuroacanthocytosis. J Mol Med 2002;80(8):475–491. 10.1007/s00109-002-0349-z. 12185448

[76] Schneider SA, Lang AE, Moro E, et al. Characteristic head drops and axial extension in advanced chorea‐acanthocytosis. Mov Disord 2010;25:1487–1491. 10.1002/mds.23052. 20544815

[77] Walker RH, Miranda M, Jung HH, et al. Life expectancy and mortality in chorea‐acanthocytosis and McLeod syndrome. Parkinsonism Relat Disord 2019;60:158–161. 10.1016/j.parkreldis.2018.09.003. 30245172

[78] Shin HW, Park HK. Recent updates on acquired hepatocerebral degeneration. Tremor Other Hyperkinet Mov 2017;7:463. 10.7916/D8TB1K44. PMC5623760 28975044

[79] Ferrara J, Jankovic J. Acquired hepatocerebral degeneration. J Neurol 2009;256:320–332. 10.1007/s00415-009-0144-7. 19224314

[80] Burkhard PR, Delavelle J, Du Pasquier R, et al. Chronic parkinsonism associated with cirrhosis. Arch Neurol 2003;60:521. 10.1001/archneur.60.4.521. 12707065

[81] Klos KJ, Ahlskog JE, Josephs KA, et al. Neurologic Spectrum of chronic liver failure and basal ganglia T1 Hyperintensity on magnetic resonance imaging. Arch Neurol 2005;62:1385. 10.1001/archneur.62.9.1385. 16157745

[82] Papapetropoulos S, Tzakis A, Sengun C, et al. Case of pediatric acquired chronic Hepatocerebral degeneration. Pediatr Neurol 2008;38:67–70. 10.1016/j.pediatrneurol.2007.09.010. 18054700

[83] Papapetropoulos S, Singer C. Management of the extrapyramidal syndrome in chronic acquired hepatocerebral degeneration (CAHD). Mov Disord 2005;20:1088–1089. 10.1002/mds.20585. 15954132

[84] Maeda H, Sato M, Yoshikawa A, et al. Brain MR imaging in patients with hepatic cirrhosis: relationship between high intensity signal in basal ganglia on T 1 ‐weighted images and elemental concentrations in brain. Neuroradiology 1997;39:546–550. 10.1007/s002340050464. 9272489

[85] Maffeo E, Montuschi A, Stura G, et al. Chronic acquired hepatocerebral degeneration, pallidal T1 MRI hyperintensity and manganese in a series of cirrhotic patients. Neurol Sci 2014;35:523–530. 10.1007/s10072-013-1458-x. 23712371

[86] Awada A, Sullivan S, Palkar V, et al. Brain magnetic resonance imaging in non‐alcoholic cirrhosis. Eur J Radiol 1995;21:84–88. 10.1016/0720-048X(95)00694-L. 8850497

[87] Krieger S, Jauss M, Jansen O, et al. Neuropsychiatric profile and hyperintense globus pallidus on T1‐ weighted magnetic resonance images in liver cirrhosis. Gastroenterology 1996;111:147–155. 10.1053/gast.1996.v111.pm8698193. 8698193

[88] Servin‐Abad L, Tzakis A, Schiff ER, et al. Acquired hepatocerebral degeneration in a patient with HCV cirrhosis: complete resolution with subsequent recurrence after liver transplantation. Liver Transpl 2006;12:1161–1165. 10.1002/lt.20815. 16799948

[89] Fichet J, Mercier E, Genée O, et al. Prognosis and 1‐year mortality of intensive care unit patients with severe hepatic encephalopathy. J Crit Care 2009;24:364–370. 10.1016/j.jcrc.2009.01.008. 19327960

[90] Dellatore P, Cheung M, Mahpour NY, et al. Clinical manifestations of hepatic encephalopathy. Clin Liver Dis 2020;24:189–196. 10.1016/j.cld.2020.01.010. 32245526

[91] Amodio P, Montagnese S, Gatta A, et al. Characteristics of minimal hepatic encephalopathy. Metab Brain Dis 2004;19:253–267. 10.1023/B:MEBR.0000043975.01841.de. 15554421

[92] Neiman J, Lang AE, Fornazzari L, et al. Movement disorders in alcoholism: a review. Neurology 1990;40:741–741. 10.1212/WNL.40.5.741. 2098000

[93] Wijdicks EFM. Hepatic encephalopathy. N Engl J Med 2016;375:1660–1670. 10.1056/NEJMra1600561. 27783916

[94] Aldridge DR, Tranah EJ, Shawcross DL. Pathogenesis of hepatic encephalopathy: role of ammonia and systemic inflammation. J Clin Exp Hepatol 2015;5:S7–S20. 10.1016/j.jceh.2014.06.004. 26041962 PMC4442852

[95] Clemmesen JO, Larsen FS, Kondrup J, et al. Cerebral herniation in patients with acute E liver failure is correlated with arterial ammonia concentration. Hepatology 1999;29:648–653. 10.1002/hep.510290309. 10051463

[96] Swaminathan M, Ellul M, Cross T. Hepatic encephalopathy: current challenges and future prospects. Hepat Med 2018;10:1–11. 10.2147/HMER.S118964. 29606895 PMC5868572

[97] Vonghia L, Leggio L, Ferrulli A, et al. Acute alcohol intoxication. Eur J Intern Med 2008;19:561–567. 10.1016/j.ejim.2007.06.033. 19046719

[98] Noble JM, Weimer LH. Neurologic complications of alcoholism. Continuum 2014;20:624–641. 10.1212/01.CON.0000450970.99322.84. 24893238 PMC10563903

[99] Yokota O, Tsuchiya K, Terada S, et al. Frequency and clinicopathological characteristics of alcoholic cerebellar degeneration in Japan: a cross‐sectional study of 1,509 postmortems. Acta Neuropathol 2006;112:43–51. 10.1007/s00401-006-0059-7. 16622656

[100] Shanmugarajah PD, Hoggard N, Currie S, et al. Alcohol‐related cerebellar degeneration: not all down to toxicity? Cerebellum Ataxias 2016;3:17. 10.1186/s40673-016-0055-1. 27729985 PMC5048453

[101] Langlais PJ. Alcohol‐related thiamine deficiency: impact on cognitive and memory functioning. Alcohol Health Res World 1995;19:113–121. 31798071 PMC6875731

[102] Koller W, O'Hara R, Dorus W, et al. Tremor in chronic alcoholism. Neurology 1985;35:1660–1660. 10.1212/WNL.35.11.1660. 4058757

[103] Jesse S, Bråthen G, Ferrara M, et al. Alcohol withdrawal syndrome: mechanisms, manifestations, and management. Acta Neurol Scand 2017;135:4–16. 10.1111/ane.12671. 27586815 PMC6084325

[104] Prakash S, Balhara YPS. Rare form of Dyskinetic movements associated with alcohol withdrawal. Indian J Psychol Med 2016;38:163–164. 10.4103/0253-7176.178816. 27114634 PMC4820561

[105] Neiman J, Borg S, Wahlund L. O. Parkinsonism and Dyskinesias during ethanol withdrawal. Addiction 1988;83:437–439. 10.1111/j.1360-0443.1988.tb00492.x. 3395725

[106] Drake ME. Recurrent spontaneous myoclonus in alcohol withdrawal. South Med J 1983;76:1040–1041. 10.1097/00007611-198308000-00028. 6879270

[107] Bijjal S, Subodh BN, Narayanaswamy JC, et al. Dystonia as a presenting feature of alcohol withdrawal. J Neuropsychiatry Clin Neurosci 2012;24:E15–E16. 10.1176/appi.neuropsych.11010026. 22450628

[108] Osna NA, Donohue TM, Kharbanda KK. Alcoholic liver disease: pathogenesis and current management. Alcohol Res 2017;38:147–161. 28988570 10.35946/arcr.v38.2.01 PMC5513682

[109] Torruellas C. Diagnosis of alcoholic liver disease. World J Gastroenterol 2014;20:11684. 10.3748/wjg.v20.i33.11684. 25206273 PMC4155359

[110] Titulaer MJ, McCracken L, Gabilondo I, et al. Treatment and prognostic factors for long‐term outcome in patients with anti‐NMDA receptor encephalitis: an observational cohort study. Lancet Neurol 2013;12:157–165. 10.1016/S1474-4422(12)70310-1. 23290630 PMC3563251

[111] Hagan H, Hepatitis C. Virus transmission dynamics in injection drug users. Subst Use Misuse 1998;33:1197–1212. 10.3109/10826089809062214. 9596383

[112] Costenbader EC, Zule WA, Coomes CM. The impact of illicit drug use and harmful drinking on quality of life among injection drug users at high risk for hepatitis C infection. Drug Alcohol Depend 2007;89:251–258. 10.1016/j.drugalcdep.2007.01.006. 17320314 PMC1974852

[113] Solomon SS, Srikrishnan AK, McFall AM, et al. Burden of liver disease among community‐based people who inject drugs (PWID) in Chennai, India. PLoS One 2016;11:e0147879. 10.1371/journal.pone.0147879. 26812065 PMC4727916

[114] Kirk GD, Astemborski J, Mehta SH, et al. Assessment of liver fibrosis by transient Elastography in persons with Hepatitis C virus infection or HIV–Hepatitis C virus Coinfection. Clin Infect Dis 2009;48:963–972. 10.1086/597350. 19236273 PMC2715996

[115] Deik A, Saunders‐Pullman R, San LM. Substance abuse and movement disorders: complex interactions and comorbidities. Curr Drug Abus Rev 2012;5:243–253. 10.2174/1874473711205030243. PMC3966544 23030352

[116] Bauer LO. Resting hand tremor in abstinent cocaine‐dependent, alcohol‐dependent, and polydrug‐dependent patients. Alcohol Clin Exp Res 1996;20:1196–1201. 10.1111/j.1530-0277.1996.tb01111.x. 8904970

[117] Duggal HS. Cocaine use as a risk factor for ziprasidone‐induced acute dystonia. Gen Hosp Psychiatry 2007;29:278–279. 10.1016/j.genhosppsych.2007.01.015. 17484950

[118] Daras M, Koppel BS, Atos‐Radzion E. Cocaine‐induced choreoathetoid movements (“crack dancing”). Neurology 1994;44:751–751. 10.1212/WNL.44.4.751. 8164838

[119] Rusyniak DE. Neurologic manifestations of chronic methamphetamine abuse. Psychiatr Clin North Am 2013;36:261–275. 10.1016/j.psc.2013.02.005. 23688691 PMC3764482

[120] de Bie RMA, Gladstone RM, Strafella AP, et al. Manganese‐induced parkinsonism associated with Methcathinone (Ephedrone) abuse. Arch Neurol 2007;64:886. 10.1001/archneur.64.6.886. 17562938

[121] Gurakar A, Tasdogan BE, Simsek C, et al. Update on immunosuppression in liver transplantation. Euroasian J Hepatogastroenterol 2019;9:96–101. 10.5005/jp-journals-10018-1301. 32117698 PMC7047305

[122] Erro R, Bacchin R, Magrinelli F, et al. Tremor induced by Calcineurin inhibitor immunosuppression: a single‐Centre observational study in kidney transplanted patients. J Neurol 2018;265:1676–1683. 10.1007/s00415-018-8904-x. 29777361

[123] Gmitterová K, Minár M, Žigrai M, et al. Tacrolimus‐induced parkinsonism in a patient after liver transplantation—Case report. BMC Neurol 2018;18:44. 10.1186/s12883-018-1052-1. 29678162 PMC5909232

[124] Wijdicks E. Neurotoxicity of immunosuppressive drugs. Liver Transpl 2001;7:937–942. 10.1053/jlts.2001.27475. 11699028

[125] Anghel D, Tanasescu R, Campeanu A, et al. Neurotoxicity of immunosuppressive therapies in organ transplantation. Maedica 2013;8:170–175. 24371481 PMC3865126

[126] Al Sibae MR, McGuire BM. Current trends in the treatment of hepatic encephalopathy. Ther Clin Risk Manag 2009;5(3):617–626. 19707277 10.2147/tcrm.s4443 PMC2724191

[127] Agarwal A, Kanekar S, Sabat S, et al. Metronidazole‐induced cerebellar toxicity. Neurol Int 2016;8:6365. 10.4081/ni.2016.6365. 27127600 PMC4830366

[128] Higashi M, Irioka T, Matsumoto T, et al. Metronidazole‐induced encephalopathy. Intern Med 2013;52:843–844. 10.2169/internalmedicine.52.9496. 23545693

[129] Andrade RJ, Aithal GP, Björnsson ES, et al. EASL clinical practice guidelines: drug‐induced liver injury. J Hepatol 2019;70:1222–1261. 10.1016/j.jhep.2019.02.014. 30926241

[130] LiverTox: Clinical and Research Information on Drug‐Induced Liver Injury [Internet]. Bethesda (MD): National Institute of Diabetes and Digestive and Kidney Diseases; 2012‐. Valproate. [Updated 2020 Jul 31]. Available from: https://www.ncbi.nlm.nih.gov/boo

[131] LiverTox: Clinical and Research Information on Drug‐Induced Liver Injury [Internet]. Bethesda (MD): National Institute of Diabetes and Digestive and Kidney Diseases; 2012‐. Tolcapone. [Updated 2017 Jul 20]. Available from: https://www.ncbi.nlm.nih.gov/boo

