# Supplementary material for: Movement Disorders and Liver Disease
Source: Mov Disord Clin Pract. 2021 May 31;8(6):828–42. doi: 10.1002/mdc3.13238 (PMC8354085; doi:10.1002/mdc3.13238)
Supplement: Supplementary file 1 — Table S1. Inherited diseases with movement disorders and liver involvement. [file MDC3-8-828-s003.docx]

**Supplementary table 1:** Inherited diseases with movement disorders and liver involvement

| **Disease**  **(inheritance)** | **Gene** | **Movement disorder/ other neurological features** | **Liver involvement/ other systemic features** | | **Additional comments** |
| --- | --- | --- | --- | --- | --- |
|  | ***Disorders of copper metabolism*** | | | | |
| Wilson’s disease  (AR) | *ATP7B* | Tremor (action, postural +/-wing-beating), dystonia (esp. face), parkinsonism, cerebellar ataxia, choreoathetosis, myoclonus, gait abnormalities.  Dysarthria, dysphagia  Psychiatric disturbances^1^ | -Asymptomatic transaminase increase, Hepatomegaly.  -Acute fulminant liver failure (F>M).  -Chronic hepatitis, cirrhosis.  -Gallstones  -Kayser-Fleischer rings, sunflower cataract, osteoporosis, haemolytic anaemia, renal stones, skin hyperpigmentation | | Brain MRI: T2 hyperintensity in basal ganglia  Treatment: copper-chelating drugs (penicillamine, trientine), zinc, liver transplantation^2^ |
|  | ***Disorders of manganese metabolism*** | | | | |
| Hypermanganesemia with dystonia-1  (AR) | *SLC30A10* | Dystonia, parkinsonism, spastic paraparesis, “cock walk” gait, postural instability^3^ | Hepatomegaly, jaundice, cirrhosis  Polycythemia | | Brain MRI: Basal ganglia+ cerebellar WM T1 hyperintensity  Treatment: disodium calcium edetate |
|  | ***Lysosomal storage disorders*** | | | | |
| Niemann-Pick disease type C  (AR) | *NPC1*  (95%)  *NPC2*  (4%) | Ataxia, dystonia ++  Parkinsonism, myoclonus, chorea. Vertical supranuclear gaze palsy, pyramidal signs, dysarthria, hearing loss; gelastic cataplexy; and seizures.  Dementia, psychiatric features^4^;Hypotonia, developmental delay *(infantile onset)*^5^ | | Transient neonatal cholestasis, hepatosplenomegaly,  liver failure *(infantile onset)*  Respiratory failure *(infantile onset)* | Brain MRI: cerebral (esp. frontal) and cerebellar atrophy^5^  Treatment: miglustat^6^ |
| Gaucher’s disease  (AR) | *GBA1* | Type 1+ heterozygotes: Parkinsonism^7^*^,^*^8^  Type 2+3: Axial rigidity, bulbar palsy, horizontal oculomotor abnormalities, pyramidal signs, myoclonic seizures, developmental delay ^9,10^ | | Hepatomegaly, focal lesions(‘Gaucheromas’), cholelithiasis, steatosis.  Late complications: cirrhosis, hepatocellular carcinoma^11^  Splenomegaly, skeletal disease, cytopenias, renal disease, pulmonary hypertension, cardiac abnormalities, higher risk of cancer^10^ | Treatment: enzyme-replacement or substrate reduction therapies (miglustat)^12^ |
| Sialidosis (mucolipidosis I)  *Type I*  *(AR)*  *Type II*  *(AR)* | *NEU1* | Progressive myoclonic epilepsy (ataxia, myoclonus, seizures)  Cognitive impairment in late stage^13^  Myoclonus, seizures, ataxia^14,15^  Developmental delay | | Hepatomegaly (mild, few cases)^16^  Visual defects (macular cherry red spots, cataracts), heart disease, hearing loss  Hepatomegaly, dysmorphic features, hearing loss | Brain MRI: cerebellar atrophy |
| G_M1_ gangliosidosis  *Type I and II (infantile and juvenile forms)*  (AR) | *GLB1* | Dystonia, ataxia.  Developmental delay, pyramidal signs, hypotonia, seizures^17,18^ | | Hepatomegaly, elevated liver enzymes.  Visceromegaly, skeletal abnormalities, macular ‘cherry red’ spot  Liver involvement generally not present in Type III (adult-onset) | Treatment: Miglustat may slow neurological^19^  Brain MRI: bilateral symmetrical hyperintense T2 signal in putamen |
| G_M2_ gangliosidosis *(*Tay–Sachs and Sandhoff disease*)*  (AR) | *HEXA*  *HEXB* | Cerebellar ataxia, dystonia, dysarthria, choreoathetosis, tremor, myoclonus  Motor neuronopathy, mental deterioration, seizures, macular ‘cherry red’ spot^20,21^ | | Hepatosplenomegaly^22,23^ | Reduced serum hexosaminidase levels suggestive |
| Prosaposin deficiency^24^  (AR) | *PSAP* | Myoclonus, tremor, dystonia, hyperkinesia.  Developmental delay, seizures, hypotonia, opisthotonus | | Hepatomegaly  Splenomegaly, microcephaly, joint contractures, optic atrophy, cataracts, respiratory insufficiency, enteropathy | Fatal within the first few months of life |
|  | ***Peroxisomal disorders***^25^ | | | | |
| PEROXISOME BIOGENESIS DISORDERS (PBD)  -Zellweger spectrum disorders (AR)  *- Zellweger syndrome*  *-Neonatal adrenoleukodystrophy (NALD)*  *- Infantile Refsum disease*  *- Heimler syndrome*  - Rhizomelic chondrodysplasia punctata Type 1 and 5 (AR)  - Peroxisomal fission defects (AD, AR)  PEROXISOMAL ENZYME DEFICIENCIES  -X-linked adrenoleucodystrophy (X-linked)  - Acyl-CoA oxidase deficiency (AR)  - D-Bifunctional protein deficiency (AR)  - 2-methyl-CoA racemase deficiency (AR)  - Refsum disease | *PEX* 1-13  *PEX7*  *DLP1*  *MFF*  *ABCD1*  *ACOX1*  HSD17B4  AMACR  *PAXH* | Complex neurological syndromes comprising variable degrees of developmental delay, failure to thrive, ataxia, dystonia, seizures, vision and hearing problems^26^.  Tremor seen in AMACR deficiency^25^.  Neurological features often more severe with peroxisome biogenesis disorders, esp. Zellweger spectrum disorders | Hepatic involvement common. Features include hepatomegaly, hypertransaminasemia, cirrhosis and liver failure. Fulminant hepatic failure possible.  Facial dysmorphism, splenomegaly and renal involvement common.  Adrenal dysfunction may be present (esp. Cerebral X-linked Adrenoleukodystrophy)^25^ | | Elevated serum VLCFA  MRI: Deep white matter and cerebellar abnormalities.  PBD may have additional malformations of cortical development |
|  | ***Disorders of lipid metabolism*** | | | | |
| Cerebrotendinous xanthomatosis  (AR) | *CYP27A1* | Ataxia, parkinsonism, dystonia, myoclonus (palatal, limb), postural tremor^27^, corticobasal syndrome^28^.  Seizures, pyramidal signs, cognitive decline, psychiatric features, peripheral neuropathy | Neonatal cholestatic jaundice, gallstones^29,30^  Diarrhoea, cataract and optic disk paleness, tendon xanthomas, cardiovascular disease, osteoporosis | | Brain MRI: cerebellar atrophy, white matter and dentate T2 hyperintensities  Treatment: bile acid supplementation |
| Abetalipoproteinaemia (Bassen-Kornzweig syndrome) and homozygous hypobetalipoproteinaemia  (AR) | *MTTP* | Cerebellar ataxia, dysarthria  Sensory ataxia, muscle weakness, peripheral neuropathy | Steatosis and elevated transaminase levels, hepatomegaly, possible progression to fibrosis, cirrhosis, hepatocellular carcinoma^31,32^.  Steatorrhea, vomiting, retinal degeneration, growth delay, anaemia | | Fat and fat-soluble vitamin (A,D,E,K) malabsorption, failure to thrive, steatorrhoea  Retinitis pigmentosa  Blood smear: Acanthocytosis  Treatment: diet and vitamin supplementation (vitamin E esp. for neurological symptoms) |
| Hereditary spastic paraplegia type 5A  (AR) | *CYP7B1* | Ataxia, dysarthria, spasticity, amyotrophy, oculomotor abnormalities, sensory loss, psychiatric disturbances^33^ | Cholestasis and liver failure in newborns. Chronic liver disease  reported in only one patient -impossible to establish a causal relationship ^34,35^ | | MRI: Cerebral white matter abnormalities; spinal cord atrophy |
| Chanarin-Dorfman syndrome (neutral lipid storage disease with ichthyosis)  (AR) | *ABHD5* | Ataxia, Myopathy, nystagmus, strabismus, mental retardation^36^ | Elevated liver enzymes, hepatosplenomegaly, steatosis, possible progression to cirrhosis  Congenital ichthyosis, cataracts, hearing loss | |  |
| Berardinelli-Seip syndrome (congenital generalized lipodystrophy type 2)  (AR) | *BSCL2* | Progressive myoclonic epilepsy^37,38^  (dystonia, ataxic gait, pyramidal signs, mental retardation, seizures) | Hepatic steatosis,  Hepatosplenomegaly  Marked lipoatrophy, insulin resistance, early-onset diabetes, cardiomyopathy, hirsutism, acanthosis nigricans, pancreatitis | | Brain MRI: progressive cortico-subcortical atrophy |
| Mevalonate kinase deficiency  (AR) | *MVK* | Cerebellar syndrome  Seizures, mental retardation^39^ | Hepatomegaly, hepatitis^40^.  Splenomegaly, lymphadenopathy, diarrhoea, skin lesions, periodic fever syndrome, cataracts, dysmorphic features | |  |
|  | ***Disorders of carbohydrate metabolism*** | | | | |
| Galactosemia (AR) | *GALT* | Ataxia, dystonia, tremor, myoclonus, stereotypies.  Dysarthria, pyramidal signs, cognitive impairment, psychiatric symptoms, epilepsy^41,42^ | Hepatocellular injury, liver failure  Neonatal period:  *E.Coli* sepsis; bleeding  Late complications:  Premature ovarian failure, cataracts | | Brain MRI: normal or atrophy; WM abnormalities  At risk populations: Irish travellers (‘classic’ galactosemia); African Americans + Black South African (‘variant’ galactosemia)  Treatment: lactose/ galactose-restricted diet^43^ |
|  | ***Congenital disorders of glycosylation*** | | | | |
| Phosphomannomutase deficiency (PMM2-CDG) (AR) | *PMM2* | Cerebellar ataxia, dystonic/dyskinetic movements, psychomotor delay, abnormal eye movements (6^th^ nerve palsy), seizures, stroke-like episodes, peripheral neuropathy^44–46^ | Hepatomegaly, increased transaminases, steatosis, liver failure  Cardiomyopathy, lipodystrophy, dysmorphism, coagulopathy, retinitis pigmentosa, endocrinopathies.  ‘Happy demeanour’ | | Liver failure seen in severe infantile phenotypes^46,47^  Brain MRI: cerebellar hypoplasia |
|  | ***Disorders of bilirubin metabolism*** | | | | |
| Crigler-Najjar syndromes I and II  (AR) | *UGT1A* | Dystonia, athetosis, spasticity, ataxia  Oculomotor impairment (upward gaze), seizures, developmental delay, cognitive impairment^48^ | Jaundice, increased transaminases, intrahepatic cholestasis, progressive fibrosis^49,50^  Hearing loss, gastrointestinal problems | | Unconjugated hyperbilirubinemia damages the CNS *(kernicterus)*  Onset in newborns  Brain MRI: basal ganglia hyperintensities  Treatment: phototherapy, liver transplantation; phenobarbital (type II)^51^ |
|  | ***Urea cycle disorders*** | | | | |
| -Argininosuccinate lyase deficiency (AR)  -Arginase deficiency (AR)  -Argininosuccinic acid synthetase deficiency (citrullinemia type 1) (AR)  -N-acetyl glutamate synthetase deficiency(AR)  -Carbamoyl phosphate synthetase deficiency (AR)  -Hyperornithinemia-hyperammonemia-homocitrullinuria (AR)  -Ornithine transcarbamylase deficiency *(most common) (X-linked)*  -Citrin deficiency (AR) | *ASL*  *ARG1*  *ASS1*  *NAGS*  *CPS1*  *SLC25A15*  *OTC*  *SLC25A13* | Episodic ataxia, tremor, confusion, seizures (hyperammonemic crises)  Newborn: lethargy, seizures, opisthotonus, coma  Later: chronic progressive neurological presentation with spastic paraplegia (esp. arginase deficiency), dystonia, ataxia, mental retardation, seizures^52–55^. 'Cerebral Palsy' mimic | Liver function tests usually increased (further so during hyperammonemic crises)  Hepatomegaly  Fibrosis and cirrhosis (especially in argininosuccinate lyase deficiency)^56^  Acute hepatitis, acute liver failure^54^  Acute liver failure common in OTC deficiency  Cirrhosis and hepatocellular carcinoma (HCC) often seen in citrullinemia type 2 or ASA^25^.  HCC can occur in the absence of cirrhosis. | | Hyperammonemia, encephalopathic crises precipitated by stress, dehydration, protein load, surgery, infection, valproate  MRI-*During crises:* Diffuse, asymmetric cerebral oedema, often involving both hemispheres and the basal ganglia +/- stroke-like lesions. Sparing of thalami, brainstem, occipital regions and cerebellum; *Chronic findings:* myelination defects, progressive cerebral atrophy[27]  Ornithine transcarbamylase deficiency: increased urinary orotic acid  Plasma arginine and citrulline levels give clue to underlying disorder  Treatment: protein-restricted diet, ammonia-reducing medication, supplementation of essential amino acids, liver transplant in selected cases |
|  | ***Organic acidurias(OA)*** | | | | |
| Prevalent Classic OA:  -Methylmalonic Aciduria (AR)  -Propionic Acidemia (AR)  -Isovaleric Acidemia (AR)  -3-Hydroxy-3-methylglutaryl-CoA lyase deficiency (AR)  -Beta-ketothiolase deficiency (AR)  Prevalent Cerebral OA:  -Glutaric academia type 1 (AR) | *MMUT, MMAA,MMAB,MMADHC,MCEE*  PCCA,PCCB  IVD  HMGCL  ACAT1  GCDH | Episodic ataxia, tremor, confusion, seizures (metabolic crises)  Later: progressive neurological syndrome with dystonia, dyskinesia, myoclonus, choreoathetosis, ataxia, seizures, mental retardation^57,58^ | Elevated liver function test, ultrasound and biopsy abnormalities  Liver cirrhosis and neoplasms (long term complications)^59,60^  Acute liver failure  Renal failure, cardiomyopathy, arrhythmias, recurrent vomiting, immunodeficiency  Glutaric aciduria type 1 may have mild derangements in liver function tests, but liver disease is often not prominent. | | Onset in newborns or later  Triggers that can precipitate a metabolic crisis: infection, fever, prolonged fasting, medication (steroids, valproate), surgery, general anaesthesia, trauma, excessive protein intake  MRI: *Glutaric aciduria type 1:* Spongiform myelinosis, widening of the sylvian fissure (“bat wing sign”) and subdural haematomas which may be mistaken as sequelae of child abuse[15,130]  Treatment: low-protein diet, ammonia-reducing medication, liver or liver/kidney transplant in selected cases |
|  | ***Aminoacidurias*** | | | | |
| Lysinuric protein intolerance(AR) | *SLC7A7* | Episodic ataxia, tremor, confusion, seizures, asterixis (hyperammonemic crises)^61^ | Hepatomegaly, cirrhosis  Interstitial pneumonia, osteoporosis, renal failure | | Protein avoidance  Can have adult onset  Treatment: low-protein diet, citrulline supplementation |
| Tyrosinemia type I  (AR) | *FAH* | Neurologic crises: Dystonic opisthotonus, acute peripheral neuropathy with painful dysaesthesias or paralysis^62^ | Acute presentations (early life): liver failure  Later presentations: Progressive liver disease, renal tubular dysfunction, hypophosphatemic rickets. Increased HCC risk. | | Treatment: nitisinone (effective if promptly initiated); Liver transplant |
|  | ***Ciliopathies*** | | | | |
| Joubert syndrome with congenital hepatic fibrosis/COACH- cerebellar vermis hypoplasia, oligophrenia, ataxia, coloboma and hepatic fibrosis- syndrome^63^  (AR) | *CC2D2A*  *TMEM67*  *RPGRIP1L* | Ataxia, dysarthria, oculomotor apraxia  Developmental delay, hypotonia^64^ | Congenital hepatic fibrosis- increased transaminases, hepatosplenomegaly, chronic hepatitis, cirrhosis  Multifocal liver tumors^65^, neonatal cholestasis^66^  Breathing abnormalities  Facial dysmorphism  Renal disease | | Brain MRI: cerebellar vermis hypo/aplasia; molar tooth sign (pathognomonic)  Other very rare ciliopathies may present cerebellar ataxia and hepatic fibrosis in a minority of cases (secondary features): Mainzer-Saldino syndrome^67^, Bardet-Biedl syndrome^68,69^, orofaciodigital syndromes^70,71^ |
|  | ***Mitochondrial disease*** | | | | |
| Hepato-cerebral mitochondrial DNA depletion syndrome  (AR) | *DGUOK*, *MPV17*, *POLG* (Alpers syndrome), *C10orf2, SUCL1G* | Ataxia, myoclonus, dystonia, choreoathetosis, parkinsonism  Psychomotor delay, nystagmus, opsoclonus, seizures, stroke-like episodes, headache, peripheral neuropathy^72^ | Cholestasis, hepatomegaly, steatosis, cirrhosis, hepatocellular carcinoma^72,73^  Lactic acidosis, sensorineural hearing impairment | | Liver failure can be precipitated by valproate^74^ |
| MEGDHEL syndrome (3-methylglutaconic aciduria with deafness, hepatopathy, encephalopathy and Leigh-like syndrome)  (AR) | *SERAC1* ^75^ | Dystonia, facial dyskinesias, spasticity  Delayed motor development, intellectual disability, hypotonia, seizures^76,77^ | Reversible neonatal liver failure. Episodic mild liver dysfunction (usually during infection), hepatomegaly  Sensorineural deafness, lactic acidosis, optic atrophy, renal tubulopathy | | Usually neonatal onset with liver failure, hypotonia and developmental delay; progressive generalized dystonia and deafness start around 18 months  Brain MRI: basal ganglia involvement with sparing of sparing of the central putamen (“putaminal eye”) |
| Pearson syndrome | mtDNA deletions | Cerebellar ataxia, tremor  Progressive external ophthalmoplegia, myopathy, seizures^78^ | Hepatomegaly, elevated liver enzymes, steatosis, hemosiderosis, cirrhosis  Bone marrow and pancreatic insufficiency, sensorineural hearing loss, renal tubular disease, diabetes mellitus, skin lesions, pigmentary retinopathy^78,79^ | | Onset in childhood and poor prognosis. Some patients survive and develop Kearns-Sayre syndrome later |
| Coenzyme Q10 deficiency syndromes (AR) | Numerous, including *COQ2-9, PDSS1 and PDSS2* | Cerebellar ataxia, dystonia  Encephalopathy, myopathy, seizures, developmental delay^80^ | Liver involvement seen especially with infantile multi-system disease, often associated with nephropathy, hearing loss, pancreatic insufficiency | | Brain MRI: cerebral/cerebellar atrophy; stroke-like lesions  Treatment: CoQ10 supplementation |
|  | ***Other heredodegenerative disorders*** | | | | |
| Ataxia telangiectasia (AR) | *ATM* | Ataxia, dystonia, chorea, tremor dopa-responsive dystonia^81^  Oculomotor apraxia | Elevated liver function tests, fatty liver, possible progression to cirrhosis^82–84^, hepatic lymphoma, hepatoblastoma^85^, hepatocellular carcinoma^86^  Ocular telangiectasias, immunodeficiency, diabetes, cancer predisposition | | Alpha-fetoprotein levels usually high |
| Neuroacahthocytosis  -Chorea-acanthocytosis (AR)  -McLeod syndrome (X-linked) | *VPS13A*  *XK* | Chorea, dystonia (esp. tongue+face^87^), secondary tics, parkinsonism, head drops and axial extension (‘rubber man’)^88^  Axonal neuropathy, myopathy, cognitive decline, psychiatric disturbances, seizures | Increased liver enzymes, hepatomegaly (esp. McLeod)^89,90,91,92^  Cardiomyopathy, arrhythmias (McLeod) ^93^ | | Elevated creatine kinase (CK)  Blood smear: acanthocytes  Brain MRI: caudate atrophy |
| Aceruloplasminemia (AR) | *CP* | Dystonia, ataxia, parkinsonism, craniofacial dyskinesias  Dementia^94^ | Hepatic iron overload (usually asymptomatic) ^95,96^, occasionally mild-moderate fibrosis on biopsy^97^  Retinal degeneration, diabetes, anaemia | | Onset in 3^rd^ or 4^th^ decade  Bloods: Low caeruloplasmin, increased ferritin  Treatment: iron chelation |
| Hereditary haemochromatosis (AR) | HFE | Ataxia, parkinsonism, tremor, myoclonus, cervical dystonia, chorea  Cognitive impariment^98,99^ | Hepatomegaly, cirrhosis, hepatocellular carcinoma^100^  Endocrinopathies, bronze skin pigmentation, cardiomyopathy, cardiac arrhythmias, arthritis, osteoporosis | | Movement disorders described in few patients - debatable whether these are causally related to systemic/brain iron overload^101,102^ . C282Y homozygosity is >0.5% of northern European caucasians^103^  Treatment: phlebotomy, liver transplant |
| Autosomal recessive spinocerebellar ataxia 21 (AR) | *SCYL1* | Ataxia, action tremor  Peripheral neuropathy, intellectual disability^104,105^ | Recurrent episodes of liver failure early in life, causing progressive fibrosis+cirrhosis.  Hepatosplenomegaly, skeletal anomalies | | Childhood Onset  Brain MRI: cerebellar atrophy |

AD: autosomal dominant; AR: autosomal recessive; CNS: central nervous system; F: female; HCC: hepatocellular carcinoma; M: male; MRI: magnetic resonance imaging; VLCFA: very long chain fatty acids; WM: white matter

**References**

1 Bandmann O, Weiss KH, Kaler SG. Wilson’s disease and other neurological copper disorders. *Lancet Neurol* 2015; **14**: 103–13.

2 Aggarwal A, Bhatt M. The Pragmatic Treatment of Wilson’s Disease. *Mov Disord Clin Pract* 2014; **1**: 14–23.

3 Stamelou M, Tuschl K, Chong WK, *et al.* Dystonia with brain manganese accumulation resulting from SLC30A10 mutations: A new treatable disorder. *Mov Disord* 2012; **27**: 1317–22.

4 Sevin M, Lesca G, Baumann N, *et al.* The adult form of Niemann-Pick disease type C. *Brain* 2006; **130**: 120–33.

5 Geberhiwot T, Moro A, Dardis A, *et al.* Consensus clinical management guidelines for Niemann-Pick disease type C. *Orphanet J Rare Dis* 2018; **13**: 50.

6 Pineda M, Walterfang M, Patterson MC. Miglustat in Niemann-Pick disease type C patients: a review. *Orphanet J Rare Dis* 2018; **13**: 140.

7 Bembi B, Zambito Marsala S, Sidransky E, *et al.* Gaucher’s disease with Parkinson’s disease: Clinical and pathological aspects. *Neurology* 2003; **61**: 99–101.

8 Blandini F, Cilia R, Cerri S, *et al.* Glucocerebrosidase mutations and synucleinopathies: Toward a model of precision medicine. *Mov Disord* 2019; **34**: 9–21.

9 Mignot C, Doummar D, Maire I, De Villemeur TB. Type 2 Gaucher disease: 15 new cases and review of the literature. *Brain Dev* 2006; **28**: 39–48.

10 Mignot C, Gelot A, De Villemeur TB. Gaucher disease. In: Handbook of Clinical Neurology, 1st edn. Elsevier B.V., 2013: 1709–15.

11 Adar T, Ilan Y, Elstein D, Zimran A. Liver involvement in Gaucher disease – Review and clinical approach. *Blood Cells, Mol Dis* 2018; **68**: 66–73.

12 Gary SE, Ryan E, Steward AM, Sidransky E. Recent advances in the diagnosis and management of Gaucher disease. *Expert Rev Endocrinol Metab* 2018; **13**: 107–18.

13 Wraith JE. Mucopolysaccharidoses and mucolipidoses. In: Handbook of Clinical Neurology. Elsevier B.V., 2013: 1723–9.

14 Caciotti A, Rocco M, Filocamo M, *et al.* Type II sialidosis: review of the clinical spectrum and identification of a new splicing defect with chitotriosidase assessment in two patients. *J Neurol* 2009; **256**: 1911–5.

15 Franceschetti S, Canafoglia L. Sialidoses. *Epileptic Disord* 2016; **18**: 89–93.

16 Caciotti A, Melani F, Tonin R, *et al.* Type I sialidosis, a normosomatic lysosomal disease, in the differential diagnosis of late-onset ataxia and myoclonus: An overview. *Mol Genet Metab* 2020; **129**: 47–58.

17 Arash-Kaps L, Komlosi K, Seegräber M, *et al.* The Clinical and Molecular Spectrum of GM1 Gangliosidosis. *J Pediatr* 2019; **215**: 152-157.e3.

18 Lang FM, Korner P, Harnett M, Karunakara A, Tifft CJ. The natural history of Type 1 infantile GM1 gangliosidosis: A literature-based meta-analysis. *Mol Genet Metab* 2020; **129**: 228–35.

19 Fischetto R, Palladino V, Mancardi MM, *et al.* Substrate reduction therapy with Miglustat in pediatric patients with GM1 type 2 gangliosidosis delays neurological involvement: A multicenter experience. *Mol Genet Genomic Med* 2020; **8**: e1371.

20 Sung AR, Moretti P, Shaibani A. Case of late-onset Sandhoff disease due to a novel mutation in the HEXB gene. *Neurol Genet* 2018; **4**: e260.

21 Barritt AW, Anderson SJ, Leigh PN, Ridha BH. Late-onset Tay–Sachs disease. *Pract Neurol* 2017; **17**: 396–9.

22 Tavasoli AR, Parvaneh N, Ashrafi MR, Rezaei Z, Zschocke J, Rostami P. Clinical presentation and outcome in infantile Sandhoff disease: a case series of 25 patients from Iranian neurometabolic bioregistry with five novel mutations. *Orphanet J Rare Dis* 2018; **13**: 130.

23 Karimzadeh P, Jafari N, Nejad Biglari H, *et al.* GM2-Gangliosidosis (Sandhoff and Tay Sachs disease): Diagnosis and Neuroimaging Findings (An Iranian Pediatric Case Series). *Iran J child Neurol* 2014; **8**: 55–60.

24 Motta M, Tatti M, Furlan F, *et al.* Clinical, biochemical and molecular characterization of prosaposin deficiency. *Clin Genet* 2016; **90**: 220–9.

25 Tan AP, Gonçalves FG, Almehdar A, Soares BP. Clinical and Neuroimaging Spectrum of Peroxisomal Disorders. *Top Magn Reson Imaging* 2018; **27**: 241–57.

26 Vilarinho S, Sari S, Mazzacuva F, *et al.* ACOX2 deficiency: A disorder of bile acid synthesis with transaminase elevation, liver fibrosis, ataxia, and cognitive impairment. *Proc Natl Acad Sci* 2016; **113**: 11289–93.

27 Stelten BML, van de Warrenburg BPC, Wevers RA, Verrips A. Movement disorders in cerebrotendinous xanthomatosis. *Parkinsonism Relat Disord* 2019; **58**: 12–6.

28 Rubio-Agusti I, Kojovic M, Edwards MJ, *et al.* Atypical parkinsonism and cerebrotendinous xanthomatosis: Report of a family with corticobasal syndrome and a literature review. *Mov Disord* 2012; **27**: 1769–74.

29 Nie S, Chen G, Cao X, Zhang Y. Cerebrotendinous xanthomatosis: a comprehensive review of pathogenesis, clinical manifestations, diagnosis, and management. *Orphanet J Rare Dis* 2014; **9**: 179.

30 Salen G, Steiner RD. Epidemiology, diagnosis, and treatment of cerebrotendinous xanthomatosis (CTX). *J Inherit Metab Dis* 2017; **40**: 771–81.

31 Lee J, Hegele RA. Abetalipoproteinemia and homozygous hypobetalipoproteinemia: a framework for diagnosis and management. *J Inherit Metab Dis* 2014; **37**: 333–9.

32 Welty FK. Hypobetalipoproteinemia and abetalipoproteinemia. *Curr Opin Lipidol* 2020; **31**: 49–55.

33 Schöls L, Rattay TW, Martus P, *et al.* Hereditary spastic paraplegia type 5: natural history, biomarkers and a randomized controlled trial. *Brain* 2017; **140**: 3112–27.

34 Goizet C, Boukhris A, Durr A, *et al.* CYP7B1 mutations in pure and complex forms of hereditary spastic paraplegia type 5. *Brain* 2009; **132**: 1589–600.

35 Lan MY, Yeh TH, Chang YY, *et al.* Clinical and genetic analysis of Taiwanese patients with hereditary spastic paraplegia type 5. *Eur J Neurol* 2015; **22**: 211–4.

36 Redaelli C, Coleman RA, Moro L, *et al.* Clinical and genetic characterization of Chanarin-Dorfman Syndrome patients: first report of large deletions in the ABHD5 gene. *Orphanet J Rare Dis* 2010; **5**: 33.

37 Opri R, Fabrizi GM, Cantalupo G, *et al.* Progressive Myoclonus Epilepsy in Congenital Generalized Lipodystrophy type 2: Report of 3 cases and literature review. *Seizure* 2016; **42**: 1–6.

38 Serino D, Davico C, Specchio N, Marras CE, Fioretto F. Berardinelli-Seip syndrome and progressive myoclonus epilepsy. *Epileptic Disord* 2019; **21**: 117–21.

39 Simon A, Kremer HPH, Wevers RA, *et al.* Mevalonate kinase deficiency: Evidence for a phenotypic continuum. *Neurology* 2004; **62**: 994–7.

40 Zhang S. Natural history of mevalonate kinase deficiency: a literature review. *Pediatr Rheumatol* 2016; **14**: 30.

41 Rubio-Agusti I, Carecchio M, Bhatia KP, *et al.* Movement Disorders in Adult Patients With Classical Galactosemia. *Mov Disord* 2013; **28**: 804–10.

42 Kuiper A, Grünewald S, Murphy E, *et al.* Movement disorders and nonmotor neuropsychological symptoms in children and adults with classical galactosemia. *J Inherit Metab Dis* 2019; **42**: 451–8.

43 Demirbas D, Coelho AI, Rubio-Gozalbo ME, Berry GT. Hereditary galactosemia. *Metabolism* 2018; **83**: 188–96.

44 Freeze HH, Eklund EA, Ng BG, Patterson MC. Neurology of inherited glycosylation disorders. *Lancet Neurol* 2012; **11**: 453–66.

45 Serrano M, de Diego V, Muchart J, *et al.* Phosphomannomutase deficiency (PMM2-CDG): ataxia and cerebellar assessment. *Orphanet J Rare Dis* 2015; **10**: 138.

46 Altassan R, Péanne R, Jaeken J, *et al.* International clinical guidelines for the management of phosphomannomutase 2‐congenital disorders of glycosylation: Diagnosis, treatment and follow up. *J Inherit Metab Dis* 2019; **42**: 5–28.

47 Jaeken J. Congenital disorders of glycosylation. In: Handbook of Clinical Neurology. Elsevier B.V., 2013: 1737–43.

48 Shapiro SM, Bhutani VK, Johnson L. Hyperbilirubinemia and Kernicterus. *Clin Perinatol* 2006; **33**: 387–410.

49 Strauss KA, Ahlfors CE, Soltys K, *et al.* Crigler‐Najjar Syndrome Type 1: Pathophysiology, Natural History, and Therapeutic Frontier. *Hepatology* 2020; **71**: 1923–39.

50 Mitchell E, Ranganathan S, McKiernan P, *et al.* Hepatic Parenchymal Injury in Crigler-Najjar Type I. *J Pediatr Gastroenterol Nutr* 2018; **66**: 588–94.

51 Bosma PJ. Inherited disorders of bilirubin metabolism. *J Hepatol* 2003; **38**: 107–17.

52 Smith W, Kishnani PS, Lee B, *et al.* Urea Cycle Disorders: Clinical Presentation Outside the Newborn Period. *Crit Care Clin* 2005; **21**: S9–17.

53 Gordon N. Ornithine transcarbamylase deficiency: a urea cycle defect. *Eur J Paediatr Neurol* 2003; **7**: 115–21.

54 Martinelli D, Diodato D, Ponzi E, *et al.* The hyperornithinemia–hyperammonemia-homocitrullinuria syndrome. *Orphanet J Rare Dis* 2015; **10**: 29.

55 Sin YY, Baron G, Schulze A, Funk CD. Arginase-1 deficiency. *J Mol Med* 2015; **93**: 1287–96.

56 Nagamani SCS, Erez A, Lee B. Argininosuccinate lyase deficiency. *Genet Med* 2012; **14**: 501–7.

57 Kölker S, Burgard P, Sauer SW, Okun JG. Current concepts in organic acidurias: understanding intra- and extracerebral disease manifestation. *J Inherit Metab Dis* 2013; **36**: 635–44.

58 Baumgartner MR, Hörster F, Dionisi-Vici C, *et al.* Proposed guidelines for the diagnosis and management of methylmalonic and propionic acidemia. *Orphanet J Rare Dis* 2014; **9**: 130.

59 Imbard A, Garcia Segarra N, Tardieu M, *et al.* Long-term liver disease in methylmalonic and propionic acidemias. *Mol Genet Metab* 2018; **123**: 433–40.

60 Forny P, Hochuli M, Rahman Y, *et al.* Liver neoplasms in methylmalonic aciduria: An emerging complication. *J Inherit Metab Dis* 2019; **42**: 793–802.

61 Camargo SMR, Bockenhauer D, Kleta R. Aminoacidurias: Clinical and molecular aspects. *Kidney Int* 2008; **73**: 918–25.

62 Chinsky JM, Singh R, Ficicioglu C, *et al.* Diagnosis and treatment of tyrosinemia type I: a US and Canadian consensus group review and recommendations. *Genet Med* 2017; **19**: 1380–1380.

63 Doherty D, Parisi MA, Finn LS, *et al.* Mutations in 3 genes (MKS3, CC2D2A and RPGRIP1L) cause COACH syndrome (Joubert syndrome with congenital hepatic fibrosis). *J Med Genet* 2010; **47**: 8–21.

64 Romani M, Micalizzi A, Valente EM. Joubert syndrome: congenital cerebellar ataxia with the molar tooth. *Lancet Neurol* 2013; **12**: 894–905.

65 Kirchner G. COACH syndrome associated with multifocal liver tumors. *Am J Gastroenterol* 2002; **97**: 2664–9.

66 Weiland MD, Nowicki MJ, Jones JK, Giles HW. COACH Syndrome: An Unusual Cause of Neonatal Cholestasis. *J Pediatr* 2011; **158**: 858-858.e1.

67 Beals RK, Weleber RG. Conorenal dysplasia: A syndrome of cone-shaped epiphysis, renal disease in childhood, retinitis pigmentosa and abnormality of the proximal femur. *Am J Med Genet Part A* 2007; **143A**: 2444–7.

68 Branfield Day L, Quammie C, Héon E, *et al.* Liver anomalies as a phenotype parameter of Bardet-Biedl syndrome. *Clin Genet* 2016; **89**: 507–9.

69 Forsythe E, Kenny J, Bacchelli C, Beales PL. Managing Bardet–Biedl Syndrome—Now and in the Future. *Front Pediatr* 2018; **6**: 1–8.

70 Rock N, McLin V. Liver involvement in children with ciliopathies. *Clin Res Hepatol Gastroenterol* 2014; **38**: 407–14.

71 Faily S, Perveen R, Chandler K, Clayton-Smith J. Oral-Facial-Digital Syndrome Type 1: Further Clinical and Molecular Delineation in 2 New Families. *Cleft Palate-Craniofacial J* 2020; **57**: 606–15.

72 El-Hattab AW, Scaglia F. Mitochondrial DNA Depletion Syndromes: Review and Updates of Genetic Basis, Manifestations, and Therapeutic Options. *Neurotherapeutics* 2013; **10**: 186–98.

73 El-Hattab AW, Wang J, Dai H, *et al.* MPV17 -related mitochondrial DNA maintenance defect: New cases and review of clinical, biochemical, and molecular aspects. *Hum Mutat* 2018; **39**: 461–70.

74 Tzoulis C. The spectrum of clinical disease caused by the A467T and W748S POLG mutations: a study of 26 cases. *Brain* 2006; **129**: 1685–92.

75 Wortmann SB, Vaz FM, Gardeitchik T, *et al.* Mutations in the phospholipid remodeling gene SERAC1 impair mitochondrial function and intracellular cholesterol trafficking and cause dystonia and deafness. *Nat Genet* 2012; **44**: 797–802.

76 Sarig O, Goldsher D, Nousbeck J, *et al.* Infantile mitochondrial hepatopathy is a cardinal feature of MEGDEL syndrome (3-Methylglutaconic aciduria type IV with sensorineural deafness, encephalopathy and leigh-Like Syndrome) caused by novel mutations in SERAC1. *Am J Med Genet Part A* 2013; **161**: 2204–15.

77 Maas RR, Iwanicka‐Pronicka K, Kalkan Ucar S, *et al.* Progressive deafness–dystonia due to SERAC1 mutations: A study of 67 cases. *Ann Neurol* 2017; **82**: 1004–15.

78 Wild KT, Goldstein AC, Muraresku C, Ganetzky RD. Broadening the phenotypic spectrum of Pearson syndrome: Five new cases and a review of the literature. *Am J Med Genet Part A* 2020; **182**: 365–73.

79 Lee W, Sokol R. Liver Disease in Mitochondrial Disorders. *Semin Liver Dis* 2007; **27**: 259–73.

80 Emmanuele V, López LC, Berardo A, *et al.* Heterogeneity of Coenzyme Q 10 Deficiency. *Arch Neurol* 2012; **69**. DOI:10.1001/archneurol.2012.206.

81 Charlesworth G, Mohire MD, Schneider SA, Stamelou M, Wood NW, Bhatia KP. Ataxia telangiectasia presenting as dopa-responsive cervical dystonia. *Neurology* 2013; **81**: 1148–51.

82 Weiss B, Krauthammer A, Soudack M, *et al.* Liver Disease in Pediatric Patients With Ataxia Telangiectasia. *J Pediatr Gastroenterol Nutr* 2016; **62**: 550–5.

83 Paulino TL, Rafael MN, Hix S, *et al.* Is age a risk factor for liver disease and metabolic alterations in ataxia Telangiectasia patients? *Orphanet J Rare Dis* 2017; **12**: 136.

84 Krauthammer A, Lahad A, Goldberg L, *et al.* Elevated IgM levels as a marker for a unique phenotype in patients with Ataxia telangiectasia. *BMC Pediatr* 2018; **18**: 185.

85 Cecinati V, Arcamone G, De Mattia D, Santoro N, Martire B. Hepatic non-Hodgkin lymphoma and hepatoblastoma complicating ataxia-telangiectasia. *Immunopharmacol Immunotoxicol* 2012; **34**: 1–3.

86 KUMAR GK, SAADI AA, YANG S, McCAUGHEY RS. Ataxia-telangiectasia and hepatocellular carcinoma. *Am J Med Sci* 1979; **278**: 178.

87 Schneider SA, Aggarwal A, Bhatt M, *et al.* Severe tongue protrusion dystonia: Clinical syndromes and possible treatment. *Neurology* 2006; **67**: 940–3.

88 Schneider SA, Lang AE, Moro E, Bader B, Danek A, Bhatia KP. Characteristic head drops and axial extension in advanced chorea-acanthocytosis. *Mov Disord* 2010; **25**: 1487–91.

89 Jung HH, Danek A, Walker RH. Neuroacanthocytosis Syndromes. *Orphanet J Rare Dis* 2011; **6**: 68.

90 Walker RH. Chorea. *Contin (Minneap Minn)* 2013; **19**: 1242–63.

91 Weaver J, Sarva H, Barone D, *et al.* McLeod syndrome: Five new pedigrees with novel mutations. *Parkinsonism Relat Disord* 2019; **64**: 293–9.

92 Danek A, Rubio JP, Rampoldi L, *et al.* McLeod neuroacanthocytosis: Genotype and phenotype. *Ann Neurol* 2001; **50**: 755–64.

93 Roulis E, Hyland C, Flower R, Gassner C, Jung HH, Frey BM. Molecular Basis and Clinical Overview of McLeod Syndrome Compared With Other Neuroacanthocytosis Syndromes. *JAMA Neurol* 2018; **75**: 1554.

94 Schneider SA, Hardy J, Bhatia KP. Syndromes of neurodegeneration with brain iron accumulation (NBIA): An update on clinical presentations, histological and genetic underpinnings, and treatment considerations. *Mov Disord* 2012; **27**: 42–53.

95 Marchi G, Busti F, Lira Zidanes A, Castagna A, Girelli D. Aceruloplasminemia: A Severe Neurodegenerative Disorder Deserving an Early Diagnosis. *Front Neurosci* 2019; **13**: 1–8.

96 Vila Cuenca M, Marchi G, Barqué A, *et al.* Genetic and Clinical Heterogeneity in Thirteen New Cases with Aceruloplasminemia. Atypical Anemia as a Clue for an Early Diagnosis. *Int J Mol Sci* 2020; **21**: 2374.

97 Pelucchi S, Mariani R, Ravasi G, *et al.* Phenotypic heterogeneity in seven Italian cases of aceruloplasminemia. *Parkinsonism Relat Disord* 2018; **51**: 36–42.

98 Demarquay G, Setiey A, Morel Y, Trepo C, Chazot G, Broussolle E. Clinical report of three patients with hereditary hemochromatosis and movement disorders. *Mov Disord* 2000; **15**: 1204–9.

99 Kumar N, Rizek P, Sadikovic B, Adams PC, Jog M. Movement Disorders Associated With Hemochromatosis. *Can J Neurol Sci / J Can des Sci Neurol* 2016; **43**: 801–8.

100 Brissot P, Pietrangelo A, Adams PC, de Graaff B, McLaren CE, Loréal O. Haemochromatosis. *Nat Rev Dis Prim* 2018; **4**: 18016.

101 Russo N, Edwards M, Andrews T, O’Brien M, Bhatia KP. Hereditary haemochromatosis is unlikely to cause movement disorders. *J Neurol* 2004; **251**: 849–52.

102 Demarquay G, Thobois S, Latour P, Broussolle E. Hereditary hemochromatosis and movement disorders: the still controversial relationship. *J Neurol* 2006; **253**: 261–2.

103 Distante S. HFE gene mutation (C282Y) and phenotypic expression among a hospitalised population in a high prevalence area of haemochromatosis. *Gut* 2000; **47**: 575–9.

104 Schmidt WM, Rutledge SL, Schüle R, *et al.* Disruptive SCYL1 Mutations Underlie a Syndrome Characterized by Recurrent Episodes of Liver Failure, Peripheral Neuropathy, Cerebellar Atrophy, and Ataxia. *Am J Hum Genet* 2015; **97**: 855–61.

105 Shohet A, Cohen L, Haguel D, *et al.* Variant in SCYL1 gene causes aberrant splicing in a family with cerebellar ataxia, recurrent episodes of liver failure, and growth retardation. *Eur J Hum Genet* 2019; **27**: 263–8.
