# Supplementary material for: Movement Disorders and Liver Disease
Source: Mov Disord Clin Pract. 2021 May 31;8(6):828–42. doi: 10.1002/mdc3.13238 (PMC8354085; doi:10.1002/mdc3.13238)
Supplement: Supplementary file 2 — Table S2. Acquired diseases with movement disorders and liver involvement. [file MDC3-8-828-s002.docx]

**Supplementary table 2:** Acquired diseases with movement disorders and liver Involvement

| **Acquired disorders** | **Movement disorder/ other neurological features** | **Liver involvement/ other systemic features** | **Additional comments** |
| --- | --- | --- | --- |
| Acquired Hepatocerebral Degeneration (AHD) | Subacute onset of rapidly progressive atypical parkinsonism with prominent ataxic gait impairment.  Dystonia-predominant phenotypes described.  Cognitive involvement common^1–4^ | Primary liver affectation variable, but AHD development often coincident with development of porto-systemic shunting | MRI Brain: T1 pallidal hyperintensity (Mn)  Elevated serum Mn levels^1^ |
| Hepatic Encephalopathy | Conscious alteration + negative myoclonus^5^ | Can occur secondary to acute hepatic failure (osmotic shifts, risk of cerebral oedema) or decompensation of chronic liver disease | Treatable – ammonia reduction therapy, management of elevated ICP^5–7^  High mortality without transplant |
| Coeliac Disease | Ataxia +/- myoclonus (especially foot)  Less common: chorea^8–10^ | Most commonly asymptomatic transaminitis (responds to GFD)^11^ | Positive TTG-IgA / gut biopsy  Improvement with GFD:  -Ataxia/myoclonus: no  -Chorea: yes |
| Alcohol-related diseases | 1. Ataxia-acute intoxication/ chronic cerebellar degeneration/ nutritional (B-vitamins)^12^ 2. Tremor- withdrawal/chronic alcoholism^12^ | Alcoholic steatosis progressing to steatohepatitis and eventually cirrhosis^13,14^ | AST>ALT |
| Illicit drugs | Movement disorder depends on substance+/- adulterants^15^:  -Cocaine: tics, dystonia, chorea  -Opioids: myoclonus, occasionally acute parkinsonism^16^  -Ephedrone: parkinsonism  -Amphetamines: tremor, ataxia | Various causes:  -Alcohol abuse  -Hepatitis virus infection (needle sharing)  -Drug/adulterant side-effect | Ephedrone: pallidal T1-hyperintensity on MRI (Mn deposition) |
| Infections | *Hepatitis Viruses:*  -Ataxia, parkinsonism described as part of encephalitic illnesses^17,18^  -Hep C is associated with development of PD^19^  *Flaviviruses* (esp. West Nile virus, Japanese Encephalitis virus):  -Parkinsonism +/- dystonia | Acute infectious hepatitis  Increased AST/ALT  Spectrum of severity-fulminant hepatic failure may occur^20^ | MRI: Double-doughnut sign with flavivirus infection^21–23^ |

AHD: acquired hepatocerebral degeneration; ALT: alanine transaminase; AST: aspartate aminotransferase; GFD: gluten-free diet; ICP: intracranial pressure; IgA: Immunoglobulin A; Mn: manganese; MRI: magnetic resonance imaging; PD: Parkinson’s disease; TTG: tissue transglutaminase

**References**

1. Maffeo E, Montuschi A, Stura G, Giordana MT. Chronic acquired hepatocerebral degeneration, pallidal T1 MRI hyperintensity and manganese in a series of cirrhotic patients. Neurol. Sci. 2014;35(4):523–530.

2. Ferrara J, Jankovic J. Acquired hepatocerebral degeneration. J. Neurol. 2009;256(3):320–332.

3. Shin HW, Park HK. Recent updates on acquired hepatocerebral degeneration. Tremor and Other Hyperkinetic Movements 2017;7:463.

4. Papapetropoulos S, Singer C. Management of the extrapyramidal syndrome in chronic acquired hepatocerebral degeneration (CAHD). Mov. Disord. 2005;20(8):1088–1089.

5. Wijdicks EFM. Hepatic Encephalopathy. N. Engl. J. Med. 2016;375(17):1660–1670.

6. Clemmesen JO, Larsen FS, Kondrup J, et al. Cerebral herniation in patients with acute E liver failure is correlated with arterial ammonia concentration. Hepatology 1999;29(3):648–653.

7. Swaminathan M, Ellul M, Cross T. Hepatic encephalopathy: current challenges and future prospects. Hepatic Med. Evid. Res. 2018;Volume 10:1–11.

8. Bhatia KP, Brown P, Gregory R, et al. Progressive myoclonic ataxia associated with coeliac disease. Brain 1995;118(5):1087–1093.

9. Jesús S, Latorre A, Vinuela A, et al. Stimulus Sensitive Foot Myoclonus: A Clue to Coeliac Disease. Mov. Disord. Clin. Pract. 2019;6(4):320–323.

10. Pereira AC, Edwards MJ, Buttery PC, et al. Choreic syndrome and coeliac disease: A hitherto unrecognised association. Mov. Disord. 2004;19(4):478–482.

11. Rubio-Tapia A, Murray JA. Liver involvement in celiac disease. Minerva Med. 2008;99(6):595–604.

12. Neiman J, Lang AE, Fornazzari L, Carlen PL. Movement disorders in alcoholism: A review. Neurology 1990;40(5):741–741.

13. Osna NA, Donohue TM, Kharbanda KK. Alcoholic Liver Disease: Pathogenesis and Current Management. Alcohol Res. 2017;38(2):147–161.

14. Torruellas C. Diagnosis of alcoholic liver disease. World J. Gastroenterol. 2014;20(33):11684.

15. Deik A, Saunders-Pullman R, San Luciano M. Substance Abuse and Movement Disorders: Complex Interactions and Comorbidities. Curr. Drug Abus. Rev. 2012;5(3):243–253.

16. Matzler W, Nagele T, Gasser T, Kruger R. Acute parkinsonism with corresponding lesions in the basal ganglia after heroin abuse. Neurology 2007;68(6):414–414.

17. Pasha SA, Pasha SA, Suhasini T, Rao DA. Hepatitis E Virus-Associated Acute Encephalitic Parkinsonism. J. Assoc. Physicians India 2018;66(3):92–3.

18. Cheung MCM, Maguire J, Carey I, et al. Review of the neurological manifestations of hepatitis E infection. Ann. Hepatol. 2012;11(5):618–622.

19. Lin W-Y, Lin M-S, Weng Y-H, et al. Association of Antiviral Therapy With Risk of Parkinson Disease in Patients With Chronic Hepatitis C Virus Infection. JAMA Neurol. 2019;76(9):1019.

20. Georges AJ, Lesbordes JL, Georges-Courbot MC, et al. Fatal hepatitis from West Nile virus. Ann. l’Institut Pasteur / Virol. 1987;138(2):237–244.

21. Sips GJ, Wilschut J, Smit JM. Neuroinvasive flavivirus infections. Rev. Med. Virol. 2012;22(2):69–87.

22. Griffiths MJ, Turtle L, Solomon T. Japanese encephalitis virus infection. In: Handbook of Clinical Neurology. 2014 p. 561–576.

23. Gould E, Solomon T. Pathogenic flaviviruses. Lancet 2008;371(9611):500–509.
