# Supplementary material for: Movement Disorders and Liver Disease
Source: Mov Disord Clin Pract. 2021 May 31;8(6):828–42. doi: 10.1002/mdc3.13238 (PMC8354085; doi:10.1002/mdc3.13238)
Supplement: Supplementary file 3 — Supplementary material S3. Methods. [file MDC3-8-828-s001.docx]

**Methods**

A PubMed literature search for English language articles published up to June 10^th^ 2020 was conducted using the following combination of Medical Subject Headings (MeSH) terms: “liver disease” and “movement disorders”, “parkinsonian disorders”, “chorea”, “athetosis”, “dystonia”, “dyskinesia”, “tremor”, “myoclonus”, “tics”, “stereotypic movement disorders”, “ataxia”. Relevant articles were selected from this search, and from the articles’ reference lists. Associations described only in single cases could have been due to chance, and were not included.
